# Infants Segment Words from Songs—An EEG Study

**DOI:** 10.3390/brainsci10010039

**Published:** 2020-01-09

**Authors:** Tineke M. Snijders, Titia Benders, Paula Fikkert

**Affiliations:** 1 Language Development Department, Max Planck Institute for Psycholinguistics, 6500 Nijmegen, The Netherlands; 2 Donders Institute for Brain, Cognition and Behaviour, Radboud University, 6500 Nijmegen, The Netherlands; p.fikkert@let.ru.nl; 3 Department of Linguistics, Macquarie University, North Ryde 2109, Australia; 4 Centre for Language Studies, Radboud University, 6500 Nijmegen, The Netherlands

**Keywords:** word segmentation, infant, speech, song, EEG, ERP, familiarity, recognition, polarity

## Abstract

Children’s songs are omnipresent and highly attractive stimuli in infants’ input. Previous work suggests that infants process linguistic–phonetic information from simplified sung melodies. The present study investigated whether infants learn words from ecologically valid children’s songs. Testing 40 Dutch-learning 10-month-olds in a familiarization-then-test electroencephalography (EEG) paradigm, this study asked whether infants can segment repeated target words embedded in songs during familiarization and subsequently recognize those words in continuous speech in the test phase. To replicate previous speech work and compare segmentation across modalities, infants participated in both song and speech sessions. Results showed a positive event-related potential (ERP) familiarity effect to the final compared to the first target occurrences during both song and speech familiarization. No evidence was found for word recognition in the test phase following either song or speech. Comparisons across the stimuli of the present and a comparable previous study suggested that acoustic prominence and speech rate may have contributed to the polarity of the ERP familiarity effect and its absence in the test phase. Overall, the present study provides evidence that 10-month-old infants can segment words embedded in songs, and it raises questions about the acoustic and other factors that enable or hinder infant word segmentation from songs and speech.

## 1. Introduction

Parents across cultures sing songs, words sung to a tune, for their infants. They sing lullabies to soothe and comfort, and they often sing play songs to try and make their babies laugh [1]. While parents initially sing for affect regulation and social engagement, they add didactic reasons around their infant’s 10th month [2]. In fact, vocabulary acquisition is one of the primary areas in which mothers expect to see progress when they participate with their one-year-old in a musical education program [3]. Could songs indeed be beneficial for vocabulary learning?

There is evidence that infants preferentially process linguistic–phonetic information from songs compared to speech: Infants of 7 and 11 months old detect changes to syllable sequences when the syllables are sung rather than spoken [4,5], and neonates can already detect syllable co-occurrences in a continuous stream if these syllables are sung rather than produced in a flat speech register [6]. However, research so far has not yet convincingly shown that infants can use actual children’s songs to learn actual language. Firstly, the songs used in previous experiments had lyrics of only four or five words and consistently paired syllables with a single pitch pattern throughout the song, thus not reflecting the lyrical and musical complexity of actual children’s songs. Secondly, the skills assessed were only partially relevant to language acquisition: While the detection of syllable co-occurrences, as tested by [6], is important to linguistic word segmentation and was associated with the participants’ vocabulary size at 18 months of age, the ability to detect changes to syllable sequences, as assessed by [4,5], may be less critical to infants’ concurrent or later language acquisition. Finally, only [4] provided the critical evidence that children could transfer the material learned from song to recognize words in speech, which is ultimately the primary modality of spoken language communication.

Therefore, the present study aims to test whether infants are able to learn linguistically relevant units from ecologically valid children’s songs, and then also transfer these units to recognition in the spoken register. Specifically, we will assess infant word segmentation from children’s songs with full lyrical and musical complexity, asking whether infants can segment word forms within songs, and subsequently recognize those word forms in speech. Moreover, we directly compare infants’ segmentation across songs and the same materials presented in speech to assess whether songs present an advantage compared to speech.

As most research on the role of input in infant language acquisition has focused on the role of speech (for reviews: [7,8]), we will first contextualize the present study by discussing the potentially beneficial and hindering effects of songs for general language acquisition in adults, children, and infants. Then, we will review the literature on infant word segmentation, the fundamental ability to extract word forms from continuous speech input, which infants acquire in their first year of life. The present study, which assesses infant word segmentation from songs, is detailed in the final section of this introduction.

Songs can be expected to provide a good source for infant language learning, considering the beneficial effects of songs as well as music more generally on later language acquisition and processing. Songs directly aid memory for verbal material in both adults [9,10] and children [11]. Those findings have inspired research into the efficacy of songs for foreign- or second-language vocabulary acquisition, with the benefits, in particular for vocabulary acquisition, extending to children in the foreign language classroom (for reviews: [12,13]). Musical training can also enhance general auditory encoding, which indirectly improves a range of language skills (for a review: [14]), including children’s speech segmentation [15], phonological abilities [16], as well as the perception of speech prosody [17] and durational speech cues [18].

The beneficial effects of songs and music for language acquisition are generally understood in terms of both emotional–attentional and cognitive mechanisms. Musical expertise fine-tunes and enhances sensitivity to the acoustic features shared by music and speech, and it also enhances auditory attention and working memory [19,20,21,22,23,24,25,26,27,28,29]. These explanations can be extended to hypothesize that songs also provide useful linguistic input for infants. Firstly, songs grab infants’ attention at least as effectively as infant-directed speech [30,31,32,33,34], and they are more effective than speech in delaying and ameliorating distress [35,36]. Secondly, songs employ many features that infants are sensitive to in their early language acquisition, including phrasing [37,38] and rhythm [39,40]. Finally, it has been proposed that some of the beneficial effects of song on infants’ well-being are a direct result of internal rhythmic entrainment [35], which is a mechanism that has also been hypothesized to be responsible for the improved encoding of linguistic material [27,28,29]. These three effects of song on infants render it likely that infants can effectively engage their speech encoding networks to learn from songs.

Nevertheless, it is not trivial that infants pick up linguistic information from songs. Firstly, infants’ speech-honed language-learning skills may not be successful when applied to the acoustic signal of songs: lyrics sung to a melody are produced with different acoustic features than regular speech [41], including a more compressed and less consistently produced acoustic vowel space [42], cf. [43]. Secondly, even adults at times mishear words in songs, both in their non-native and native language [44,45,46]. Finally, even if infants learn words in songs, they may not be able to recognize these words in speech, the modality that is overwhelmingly used for spoken language communication. For example, the developmental literature on word segmentation shows that infants’ ability to transfer the recognition of a learned word to a new type of acoustic stimulus slowly emerges in the second half of infants’ first year. The ability to generalize across speakers, genders, or emotions emerges around 10.5 months of age [47,48], with evidence of infants’ ability to generalize across accents emerging around their first birthday [49,50]. Related work on infant word recognition suggests that infants between 8 and 10 months old might be particularly negatively impacted by speech variation [51]. Thus, it is conceivable that the infants up to one year of age are not yet able to transfer words learned from song to speech. This would pose clear boundaries to the effectiveness of songs for language acquisition in the first year of life.

The present study assesses the efficacy of songs for infant language learning through a word segmentation paradigm, testing word segmentation within songs and speech as well as subsequent generalization to speech. Segmentation, i.e., extracting individual word forms from the continuous speech stream, in which word boundaries are not typically marked by pauses, presents a sensible starting point for this research agenda, as adults find songs easier to segment than speech [52]; musical expertise is associated with better and faster segmentation in adults [53,54,55,56,57]; and musical training facilitates word segmentation in children [15]. Moreover, segmentation is critical to successful language acquisition, as the vast majority of words spoken to infants appear in continuous speech [58,59], even if parents are instructed to teach their infant a word [60,61]. Once infants have extracted word forms from the speech stream, they can more easily associate these with their meaning and thus start building a lexicon [62,63,64,65]. Word segmentation is also important in developing the language-ready brain, with word segmentation skills in infancy predicting language ability in the toddler years [6,66,67,68,69,70], although possibly not beyond [70]. Although the group-level effects of word segmentation are not always replicated [71,72], which is a topic that we will return to in the discussion, infants’ ability to segment words from continuous speech is well established (see [73] for a meta-analysis). In the present study, we ask whether songs provide one source of information in infants’ input from which they could segment words and start building their lexicon.

Infants rely heavily on language-specific rhythmic cues for word segmentation, with English-learning 7.5-month-olds relying on the strong–weak trochaic word stress, a rhythmic property, to segment words [74,75,76,77,78]. This metrical segmentation strategy is also developed by infants learning Dutch, another language with trochaic lexical stress [79], albeit at a slower rate compared to their English-learning peers [76], but not by infants learning French, a language without lexical stress [80,81,82].

Infant word segmentation is facilitated by the exaggeration of prosodic cues on the target word and across the entire speech stream. Prosodic accentuation on the target word is essential for segmentation by 6-month-olds and facilitates segmentation for 9-month-olds, although it may become less important when infants are 12 months of age [83]. Moreover, exact alignment of the accentuated pitch peak with the stressed syllable of the word appears to be critical [84]. General prosodic exaggeration across the speech stream, as observed in infant-directed speech (IDS), facilitates segmentation on the basis of transitional probabilities in 8-month-olds [85] and possibly even newborns [86]. In addition, word segmentation is easier from natural speech than from prosodically exaggerated speech [72], although the extent of the beneficial effect of IDS is still under investigation [87].

Considering that infants strongly rely on rhythmic cues and that prosodic exaggeration facilitates segmentation, it is conceivable that the clear musical rhythm and melodic cues of songs will enable and possibly facilitate infants’ speech segmentation. The aforementioned study by François and colleagues [6] has provided the first support for this hypothesis by showing that newborns are only able to extract words from an artificial speech stream that is musically enriched. However, every syllable in these “songs” was paired with a consistent tone, resulting in a song that presented each of the four tri-syllabic words with its own unique tune throughout. Therefore, it is still an open question whether infants can segment words from songs with the full melodic and lyrical complexity of actual children’s songs. Moreover, infants’ aforementioned difficulties generalizing segmented words across speakers, accents, and emotions raise the question of whether they will be able to recognize words segmented from song in speech. The present study aims to address these issues by testing whether infants can segment words from realistic children’s songs and subsequently recognize those words in continuous speech. In addition, it will assess how infants’ segmentation from song compares to their segmentation from continuous speech.

The present study employed an electroencephalography (EEG) familiarization paradigm for word segmentation [88,89]. This procedure is adapted from the behavioral two-step familiarization-then-test procedure, which first familiarizes infants with words and then tests their word recognition by comparing the (head turn) preference for speech with the familiarized target versus a novel control [90]. The EEG version of this paradigm exposes infants to a series of familiarization-then-test blocks and assesses word recognition on each block by comparing event-related potentials (ERPs) to familiarized targets and matched novel control words in the test phase. The EEG paradigm was preferred, as previous research has found it to be more sensitive than the behavioral method to (emerging) segmentation abilities [69,80,91,92]. Moreover, the EEG paradigm can uniquely reveal the time-course of the developing word recognition by comparing ERPs to the first and last target occurrences within the familiarization phase [89]. For example, tracking this temporal development of recognition in the EEG has revealed faster segmentation by newborns from a musically enriched compared to a monotonous speech stream [6].

**Figure 1:**
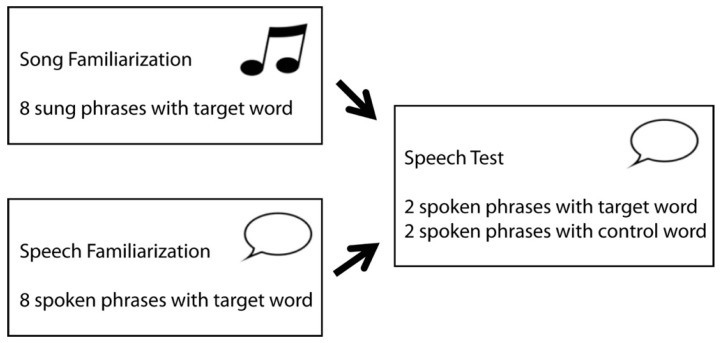
Setup of one experimental block of the study design.

The setup of the present study, as illustrated in Figure 1, was adapted from Junge and colleagues [89], who presented continuous speech in both the familiarization and test phase. Each block in the current study familiarized infants with a word embedded eight times within a sung or spoken fragment. Within this familiarization phase, the comparison of ERPs to the first two and last two occurrences of the target word enabled us to assess infants’ ability to segment words from songs and contrast it with their ability to segment words from speech. After each sung or spoken familiarization phase, infants were presented with a spoken test phase consisting of two spoken phrases with the familiarized target word, and two others with a matched novel control word. The difference in ERP response to familiarized target and novel control words after song familiarization would index infants’ ability to transfer words that are segmented from song to recognition in speech.

ERP responses to familiarized compared to novel words are generally largest over left-frontal electrode sites, and can be either positive- or negative-going, with a negativity being considered a more mature response (see review and discussion by [71,83]). The polarity of infants’ ERP familiarity response partly depends on stimulus difficulty, with 7-month-olds displaying positive-going responses to words embedded in speech after having negative-going responses to those same words presented in isolation [69]. The polarity of the response to stimuli of the same difficulty also changes developmentally, shifting from a positive-going response typically displayed by 6-month-olds to-7-month-olds [69,83] to a negative-going response after 8 months of age [67,79,83,88,92,93]. However, group effects in polarity are not consistently observed across studies due to large individual differences within age bands [71]. This variation between infants appears to be significant to early language development, as the negative responders have more robust neural segmentation responses across various stages of the procedure [70,93], better concurrent vocabulary size [71,93], better and faster vocabulary development into toddlerhood [67,69,93], and better general language skills [69].

The general developmental shift from an initial positivity to a later negativity for word recognition responses has been ascribed to cortex maturation [83] (see also [94] for a similar reasoning on the polarity of the infant MMN for auditory discrimination), as the auditory cortex undergoes tremendous changes, specifically around 6 months of age [95,96], which can influence the polarity of ERP components [97]. However, the stimulus-dependent polarity shift within a single group of infants [69] reveals that a more functional explanation is required. The polarity of an ERP depends on the location and orientation of the underlying brain activation [98]. Männel and Friederici have proposed slightly different origins of the positive and negative ERP familiarity effects (secondary auditory cortices versus superior temporal cortex, respectively), with more lexical processing due to the infants’ advancing linguistic experience resulting in the shift to superior temporal cortex activation [83]. In a similar vein, Kidd and colleagues have proposed that the negativity reflects the emergence of a lexicon [71]. We would argue similarly that the negative ERP familiarity effect reflects active lexical learning, and we interpret the negative familiarity effect as a ‘repetition enhancement’ effect (see below). When adults hear repetitions of words within sentences, a positive ERP repetition effect is elicited [99]. This can be interpreted as ‘repetition suppression’, which is a reduced neural response when a stimulus is repeated [100,101]. The positive infant ERP familiarity effect might reflect a similar repetition suppression response but now for low-level acoustic properties of the stimulus. However, the negative ERP familiarity effect might reflect ‘repetition enhancement’-enhanced processing when a stimulus is repeated [102]. Repetition enhancement effects are thought to reflect a neural learning mechanism for building or strengthening novel neural representations [103,104]. This reasoning would support the notion of the negative infant ERP familiarity effect reflecting the active building of a lexicon, which is in accordance with the proposals of [83,71].

The present study tested word segmentation in 10-month-old Dutch infants, for whom a negative-going ERP familiarity response can generally be expected in speech [67,79,88,89]. For the speech sessions, we expect to replicate the negative ERP familiarity response seen in the work by Junge and colleagues [89]. Within the song familiarization, a left frontal negative-going response to the last two compared to the first two target occurrences would be taken as evidence that word segmentation from a song is unproblematic for infants. Both negative and positive ERP familiarity responses indicate that the repetition of the word form has been identified within the continuous speech stream. However, given the previous literature, a positive response would be interpreted as indicating difficulties with song segmentation. Within the subsequent spoken test phase, a negative-going response would similarly be interpreted as automatic generalization from song to speech, with a positivity indicating a more challenging transfer process.

## 2. Materials and Methods

### 2.1. Participants

Forty Dutch 10-month-old infants participated in two experimental sessions, resulting in eighty datasets (40 song, 40 speech). The number of participants tested was based on [89]. All infants were born in term (37–42 weeks gestational age), normally developing, without a history of neurological or language impairments in the immediate family. Twenty-one datasets were excluded from analysis because of too few artefact-free EEG trials (see below). One participant was excluded because he was raised bilingually. The remaining 57 included datasets came from 32 subjects, with 25 of them contributing good data in both the speech and the song session. The 32 included subjects (16 female) were all monolingual Dutch infants (session 1: mean age 299 days, range 288–313 days; session 2: mean age 306 days, range 293–321 days). Infants were recruited from the Nijmegen Baby and Child Research Center Database. The study was approved by the local ethics committee, and parent(s) gave written informed consent for their infants prior to the experiment, in accordance with the Declaration of Helsinki.

### 2.2. Materials

**Figure 2:**
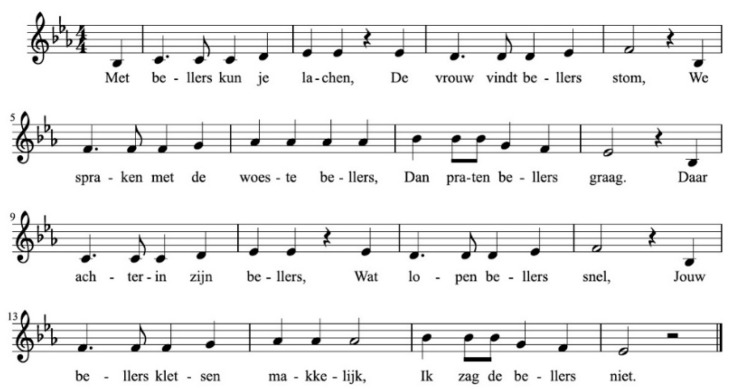
Example of score for the song that was used for target word ‘bellers’.

The familiarization materials were 20 verses of eight phrases. Each verse contained one repeating target word in every phrase (see Table 1 and Figure 2 for an example). The verses were recorded in a sung and a spoken version, for the “song familiarization” and “speech familiarization”, respectively. The “song” and “speech” stimuli used identical verses/lyrics, but only the “song” versions were recorded with the designated melodies. Each song and speech version of a verse was recorded with two different target words, for a total of four recordings per verse. The reader is referred to Supplementary Table S3 for the full set of materials.

The 20 melodies for the “song” versions all consisted of eight phrases, or of four melodic phrases that were repeated twice (with different lyrics). The melodies were (variations on) melodies of German, English, French, Norwegian, and Dutch children’s songs and unknown to a sample of 22 native Dutch parents with a 10-month-old infant (see Supplementary Table S2). The 20 original target words in [89] were supplemented with three further target words from [79,88] and 17 new target words. New target words were added to avoid the repetition of target words across blocks. All 40 target words were low-frequency trochees (see Table 2), each with a CELEX frequency lower than 19 per million [105]. The 40 target words were combined into 20 word pairs that shared a semantic category (e.g., “emoe” and “hinde”; English: emu and doe; see Table 2). Yoked pairs were created between the 20 melodies and the 20 target word pairs (Table 2). The verses that were written to each melody were made with both target words of the word pair.

The verses had a mean phrase length of 5.71 words (range: 3–10) and 7.82 syllables (range: 4–14). All eight phrases of a verse contained the target word. The target word was never the first word of a phrase, and the target word occurred maximally twice per verse in final phrase position. With one exception due to experimenter error, target words were never the last word of the first, second, seventh, or eighth sentence. The word preceding the target word was unique across the eight phrases. The main word stress of the target word consistently matched the meter of the melody in the phrase, and the text setting of the phrases to the melodies was correct, which is a condition that is considered critical for learning from songs [24]. We also created four test sentences per target word pair. The position of the target words was never the first or last of a test sentence and was otherwise variable.

Stimuli were recorded in a sound-attenuated booth, using Adobe Audition. The stimuli were annotated and further processed in Praat [106]. All stimuli were recorded by a trained singer (mezzo-soprano), and sung and spoken in a child-directed manner. The verses for the familiarization phase were typically recorded in one take per verse. Those original recordings were kept intact in terms of the order of the phrases, the duration of the phrase intervals, and the speaker’s breathing in those intervals. Three speech and one song stimuli were created by combining multiple takes to obtain stimuli without disturbing noises. Recording in one take was required to render naturally sounding song versions. In this respect, our stimulus creation is different from that used in [89], who recorded their sentences in a randomized order and combined them after the fact.

The spoken test sentences for the test phase were recorded in one take per target word pair, extracted individually from the original recordings, and played back in a randomized order in the experiment.

Finally, the attentional phrase “Luister eens!” (“Listen to this!”), which was used as a precursor to each training and test stimulus in the experiment, was recorded in both song and speech versions.

Supplementary information about the materials is given in Supplementary Table S1, with acoustic properties (duration, pitch, and loudness measures) given in Supplementary Table S1 (for phrases) and Supplementary Table S2 (for target words). The mean ‘focus’ is also reported in Supplementary Table S2, which approaches 1 if the target word is always the highest or loudest in the phrase, thus measuring acoustic prominence. Acoustic properties of the target words in phrases one and two versus those in phrases seven and eight of the familiarization stimuli were matched (see Supplementary Table S3).

### 2.3. Procedure

Infants participated in a separate song and speech session. The session order was counterbalanced across infants, with on average 7.6 days between sessions (range 5–14 days). Before the experiment started, the child could play on a play mat to get accustomed to the lab environment while the experimental procedure was explained to the parent. The EEG cap was pregelled to minimize the setup time. Then, the cap was fitted, electrode impedances were checked, and some extra gel was added where necessary. Next, the infants were seated on their parent’s lap in a sound-attenuated booth with Faraday cage, and data collection was initiated. Sung and spoken sentences were presented to the infant over two loudspeakers at 65 dB. While listening to the sentences, the infant watched a silent screen-saver (not linked to auditory input) or played with silent toys. One experimenter sat next to the screen to maintain the engagement of the infant with silent toys or soap bubbles if necessary. Both parent and experimenter listened to masking music over closed headphones. A second experimenter ran the EEG acquisition from outside the experimental booth and monitored the infant through a closed-circuit video. The experiment was stopped if the infant became distressed. One full experimental session (including preparations and breaks) took about one hour, with the experiment proper taking about 20 min.

Stimuli were presented using Presentation software [107]. In each session, the infants listened to 20 blocks of familiarization-and-test trials, with a different melody and target word pair in every block. Each block consisted of a familiarization phase immediately followed by the corresponding test phase (see Figure 1; see Figure 2 and Table 1 for an example). The eight phrases of the verse in the familiarization phase, all containing the target word, were spoken (speech session) or sung (song session). The four sentences in the test phase were always spoken: two sentences contained the ‘familiarized’ word and two contained the second ‘control’ word of the word pair, presented in a randomized order. To reduce the effects of modality switching, the attentional phrase “Luister eens!” (English: Listen to this!) was played to the infants in the modality of the session before each familiarization block. The words “Luister eens!” were always presented in the spoken modality before each test phase.

The order of the blocks was counterbalanced across subjects. Within each session, every target word was the ‘familiarized’ word for half of the infants and the ‘control’ word for the other half, and this assignment of words was counterbalanced across subjects. For each infant, the familiarized words in the spoken session were the control words in the sung session and vice versa. Note that in [89], this repetition of critical words already occurred in the second half of the experiment, which was why we accepted a repetition in the second session (after 5–14 days). The order of the blocks was such that the target words never started more than twice in a row with vowels or with the same consonantal manner or place of articulation.

### 2.4. EEG Recordings

EEG was recorded from 32 electrodes placed according to the International 10–20 system, using active Ag/AgCl electrodes (ActiCAP), Brain Amp DC, and Brain Vision Recorder software (Brain Products GmbH, Germany). The FCz electrode was used as the on-line reference. Electro-oculogram (EOG) was recorded from electrodes above (Fp1) and below the eye, and at the outer canthi of the eyes (F9, F10). The recorded EEG electrodes were F7, F3, Fz, F4, F8, FC5, FC1, FC2, FC6, T7, C3, Cz, C4, T8, TP9, CP5, CP1, CP6, TP10, P7, P3, Pz, P4, P8, PO9, and Oz. The data were recorded with a sampling rate of 500 Hz and were filtered on-line with a time constant of 10 s and a high cutoff at 1000 Hz. Electrode impedances were typically kept below 25 kΩ.

### 2.5. Data Processing

EEG data were analyzed using Fieldtrip, an open source MATLAB toolbox (The MathWorks, Natick, MA, USA) for EEG and MEG analyses [108].

First, eye movement components and noise components in the EEG data were identified using independent component analysis (ICA, [109]). In order to identify components based on as much data as possible, prior to ICA analysis, all the EEG data of the whole session were filtered from 0.1 to 30 Hz and cut in 1 s segments. Bad channels were removed, as were data segments with flat channels or large artifacts (>150 µV for EEG channels, >250 µV for EOG channels). Then, we applied infomax ICA [110] as implemented in EEGlab [111]. A trained observer (T.M.S.) identified components that revealed eye components or noise on individual electrodes. Subsequently, time-locked data were made from the original EEG data by cutting the raw data in trials from 200 ms before to 900 ms after the onset of the critical words. Again, these data were filtered from 0.1 to 30 Hz, and the bad channels were removed, as well as trials with flat channels. Then, the identified eye and noise components were removed from the time-locked data. For the included datasets (see the end of this subsection), the mean number of removed eye and noise components was 2.8 and 2.7, respectively (range: 1–5 for eye components, 0–6 for noise components). After ICA component rejection, EEG channels were re-referenced to the linked mastoids. Electrodes PO10, Oz, and PO9 were discarded, because these were bad channels for too many infants. A baseline correction was applied in which the waveforms were normalized relative to the 200 ms epoch preceding the onset of the critical word, and trials containing EEG exceeding ±150 µV were removed. Six datasets were discarded because of too many (>4) bad channels, and 15 datasets were discarded because fewer than 10 trials per condition (<25%) remained after artifact rejection. For the remaining datasets (32 subjects, 57 datasets; 31 speech, 26 song; 29 first session, 28 second session; 25 subjects with good data in both sessions), bad channels were repaired using spherical spline interpolation ([112]; mean of 0.9 channels repaired, range 0–3). Finally, ERPs were made by averaging over relevant trials. For the analyses on the combined datasets of the speech and song sessions (see below), the trials were concatenated across sessions before averaging. The combined datasets had an average of 48 included trials per condition (range 16–69), while for the single sessions, this was 26 (range 12–36) for song and 28 (range 13–36) for speech.

### 2.6. Planned ERP Analyses

For the familiarization phase, the ERP familiarity effect was assessed by comparing the ERP in response to the last two (seventh/eighth) versus the first two (first/second) target occurrences. For the test phase, the ERP familiarity effect was assessed by comparing the ERP in response to familiarized target words versus novel control words.

Analyses were performed first for the combined song and speech sessions (32 subjects). In a second step, differences between song and speech sessions were assessed, this time only including the 25 subjects that had >10 trials per condition in both sessions.

Analyses to assess the ERP familiarity effect were performed both on predefined time windows and left-frontal electrodes (based on previous literature), as well as on all time points or electrodes in a single test (to assess possible deviations from previous literature and considering the novel inclusion of the song modality).

Time windows of interest (250–500 ms and 600–800 ms) were defined based on previous literature reporting the infant ERP familiarity effect (see [71]). The left-frontal region of interest was defined as electrodes F7, F3, and F5, which are consistently included in the calculation of average amplitudes in previous literature [67,70,71,83,88,89].

The average ERPs of the left frontal region for the two time windows were assessed using SPSS, with a 2 × 2 repeated measures ANOVA on mean left-frontal ERP amplitude, with familiarity (familiar, novel) and time window (250–500, 600–800) as within-subject factors. For the modality comparison (on the 25 subjects who had good data in both sessions), modality (song, speech) was added as a within-subjects factor. For the ANOVAs, we used the Huynh–Feldt epsilon correction and reported original degrees of freedom, adjusted *p*-values, and adjusted effect sizes (partial eta-squared: *ηp*^2^).

Additionally, to explore the possible effects outside the regions and time windows of interest, ERP familiarity effects were assessed using cluster randomization tests [113]. Cluster randomization tests use the clustering of neighboring significant electrodes or time points to effectively control for multiple comparisons, while taking the electrophysiological properties of EEG into account. In these tests, first, all the electrodes or all time points are identified that exceed some prior threshold (in this case, a dependent samples *t-*test was conducted and the *p*-value was compared to a threshold alpha level of 0.05, uncorrected for multiple comparisons). In a second step, clusters are made in which neighboring electrodes or time points that exceed the threshold are grouped. For every identified cluster, a cluster-level statistic is calculated, summing the *t-*statistics of all the included electrodes/time points. A reference randomization null distribution of the maximum cluster-level statistic is obtained by randomly pairing data with conditions (e.g., familiarized and novel conditions) within every participant. This reference distribution is created from 1000 random draws, and the observed cluster *p-*value can then be estimated as the proportion from this randomization null distribution with a maximum cluster-level test statistic exceeding the observed cluster-level test statistic (Monte Carlo *p-*value). Thus, the cluster randomization *p-*value denotes the chance that such a large summed cluster-level statistic will be observed in the absence of an effect (see [113]). Note that although the observed clusters provide some information about latency and location, the cluster statistic does not define the actual spatial and temporal extent of the effect, as time points and electrodes are included based on uncorrected statistics (see [114]). Thus, precise cluster onset and extent should not be over-interpreted.

For our purposes, three exploratory cluster randomization tests were performed: two assessing all the electrodes in the two time windows (for both time windows comparing the condition averages of the time window for all electrodes and then clustering over electrodes), and one assessing all the time points from 100 to 900 ms in the left-frontal region of interest (comparing the condition averages of the three left-frontal electrodes for all time points, and then clustering over time points, starting from 100 ms after stimulus onset, as no effects were expected to occur before this time).

## 3. Results

### 3.1. Planned Analyses—Segmentation from Song and Speech

#### 3.1.1. ERP Familiarity Effect in the Familiarization Phase, Song and Speech Combined

**Figure 3:**
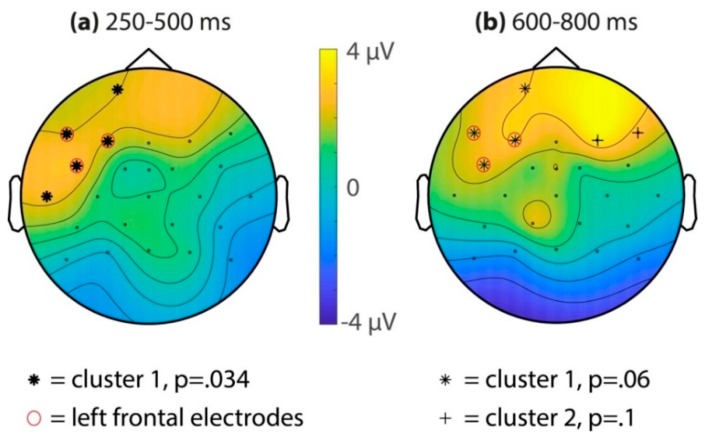
Familiarity effect in the familiarization phase for the combined sessions (32 subjects). Topographic isovoltage maps of the difference between the last two target occurrences (7th/8th) and the first two target occurrences (1st/2nd), in the (**a**) 250–500 ms and (**b**) 600–800 ms latency ranges. Electrodes that are part of output clusters in the cluster randomization test are shown with stars. The red circles indicate electrodes that are part of the left-frontal region of interest.

**Figure 4:**
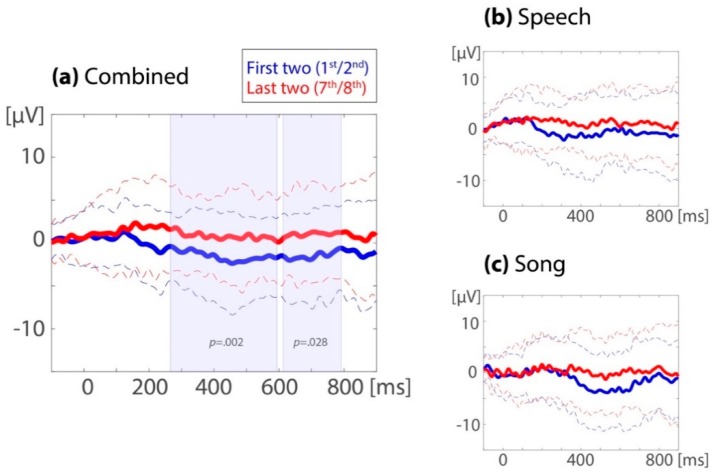
Familiarity effect in the familiarization phase. Event-related potentials (ERP) averaged over left-frontal electrodes for (**a**) the combined sessions (32 subjects), (**b**) the speech sessions (31 subjects), and (**c**) the song sessions (26 subjects). The solid lines are the ERPs from the first two target occurrences (1 and 2, in blue) and the last two target occurrences (7 and 8, in red). The means ±1 SD are given as dotted lines. The shaded areas indicate the clusters identified in the cluster randomization test.

At the left-frontal region of interest, the repeated measure ANOVA on the 32 combined datasets showed an ERP familiarity effect (*M(SD)*_unfamiliar12_ = −1.44(0.67), *M(SD*)_familiarized78_ = 0.97(0.64); *F*_familiarity_(1,31) = 11.8, *p* = 0.002, *ηp*^2^ = 0.28) with no detected difference across time windows (250–500 ms: *M(SD)*_unfamiliar12_ = −1.41(0.69), *M(SD)*_familiarized78_ = 0.93(0.65); 600–800 ms: *M(SD)*_unfamiliar12_ = −1.47(0.74), *M(SD)*_familiarized78_ = 1.00(0.76); *F*_familiarity x time window_(1,31) = 0.05, *p* = 0.83, *ηp*^2^ = 0.002). As can be seen in Figure 3 and Figure 4, the ERP was more positive for the last two (seventh/eighth) than for the first two (first/second) target occurrences.

Subsequently, all the electrodes were assessed using a cluster-randomization test, comparing the average ERP amplitudes for the last two (seventh/eighth) and first two (first/second) target occurrences for both time windows of interest (see Figure 3). For the 250–500 ms time window, this resulted in a significant cluster (*p* = 0.034) of left-frontal electrodes (Fp1, F7, F3, FC5, and T7). A second cluster over the right hemisphere (F4) did not survive multiple comparison correction (cluster *p* = 0.52). For the 600–800 ms time window, a positive cluster over the left-frontal electrodes was marginally significant (cluster *p* = 0.06, electrodes Fp1, F7, F3, and FC5). Two other clusters did not survive multiple comparisons (cluster 2: F4 and F8, *p* = 0.10; cluster 3: CP1, *p* = 0.48).

To assess all time points between 100 and 900 ms after target onset, a cluster randomization was performed on the mean of the left-frontal region (electrodes F7, F3, and F5, see Figure 4). This resulted in two significant clusters, the first ranging from 268 to 594 ms (cluster *p* = 0.004) and the second ranging from 612 to 792 ms (cluster *p* = 0.028).

The clusters identified in the cluster randomization tests match the timing and topography of the previously reported infant ERP familiarity effect. However, note that the present ERP familiarity effect is a positive shift, while previous literature on segmentation by infants of the same age has most often reported a negative shift [79,88,89]. This discrepancy will be discussed in detail in the Discussion section.

#### 3.1.2. ERP Familiarity Effect in the Familiarization Phase, Comparing Song to Speech

As can be seen in Figure 4, the ERP familiarity effects in the familiarization phase were similar across the song and speech modalities, although this effect occurred possibly somewhat later in the songs. For the 25 subjects that contributed enough data for both the speech and song sessions, the left-frontal ERP familiarity effect during the familiarization phase was compared between modalities. The repeated measures ANOVA showed a main effect of familiarity (*M(SD)*_unfamiliar12_ = −1.23(0.76), *M(SD)*_familiarized78_ = 0.32(0.71); *F*_familiarity_ (1,24) = 4.14, *p* = 0.053, *ηp^2^* = 0.15). There was no significant difference in the ERP familiarity effect between modalities (speech: *M(SD)*_unfamiliar12_ = −0.74(1.19), *M(SD)*_familiarized78_ = 0.42(0.80); song: *M(SD)*_unfamiliar12_ = −1.72(1.18), *M(SD)*_familiarized78_ = 0.23(1.10); *F*_familiarity x modality_ (1,24) = 0.13, *p* = 0.72, *ηp^2^* = 0.005), and no significant interaction between familiarity, modality, and time window (*F*_familiarity x modality x time window_ (1,24) = 0.14, *p* = 0.71, *ηp^2^* = 0.006).

Cluster-randomization tests were performed to explore the possible modality differences in ERP familiarity effects outside the regions and time windows of interest. First, all electrodes were assessed in the 250–500 ms as well as the 600–800 ms time window, comparing the difference between the first two (first/second) and last two (seventh/eighth) target occurrences across the speech and the song modality. No clusters were identified, meaning that not one electrode showed a significant interaction between modality and familiarity, even prior to corrections for multiple comparisons. Then, all time points were assessed in the left-frontal region of interest, comparing the familiarity effect across modalities. Again, no clusters were identified, meaning that not one time point showed a significant interaction between modality and familiarity, even when uncorrected for multiple comparisons. In sum, no differences could be identified in the ERP familiarity effect between the song and the speech modality, and there was no evidence for an earlier start of the ERP familiarity effect in speech.

### 3.2. Planned Analyses Effects in Test Phase (Transfer to Speech)

#### 3.2.1. ERP Familiarity Effect in the Test Phase, Song and Speech Combined

**Figure 5:**
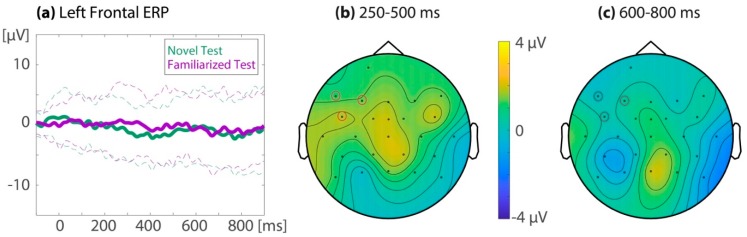
Familiarity effect in the test phase for the combined sessions (32 subjects). (**a**) Event-related potentials (ERP) averaged over left-frontal electrodes (red circles in (**b**) and (**c**)). The solid lines are the ERPs from the novel control words in the test phase (in green) and the familiarized target words in the test phase (in purple). The means ±1 SD are given as dotted lines. Right: Topographic isovoltage maps of the difference between the familiarized target words and novel control words during test, in the (**b**) 250–500 ms and (**c**) 600–800 ms latency ranges. The red circles indicate electrodes that are part of the left-frontal region of interest.

Figure 5 displays the ERPs for familiarized target words and novel control words in the test phase, as well as the topography of their difference. For the left-frontal region of interest, the repeated measures ANOVA showed no difference between the ERP to the familiarized and the novel test items (*M(SD)*_novelTest_ = −1.32(0.73), *M(SD)*_familiarizedTest_ = −0.51(0.80); *F*_familiarity_ (1,31) = 0.80, *p* = 0.38, *ηp*^2^ = 0.025), with no difference in the ERP familiarity effect between time windows (250–500 ms: *M(SD)*_novelTest_ = −1.49(0.68), *M(SD)*_familiarizedTest_ = −0.07(0.84); 600–800 ms: *M(SD)*_novelTest_ = −1.16(0.89), *M(SD)*_familiarizedTest_ = −0.95(0.92); *F*_familiarity x time window_ (1,31) = 1.69, *p* = 0.20, *ηp*^2^ = 0.05).

When assessing the ERP familiarity effect during test on all electrodes using the cluster randomization test, one effect was identified at T7 in the 250–500 ms time window, which did not survive multiple comparisons correction (cluster *p* = 0.32). No clusters were identified in the 600–800 ms time window. When assessing all time points between 100 and 900 ms for the left-frontal region of interest, one cluster was identified from 330 to 350 ms, which did not survive multiple comparisons (cluster *p* = 0.26). Thus, no significant ERP familiarity effect could be identified in the test phase.

#### 3.2.2. ERP Familiarity Effect in the Test Phase, Comparing Song to Speech

To assess the possible differences in ERP familiarity effect in the test phase across modalities, a repeated measures ANOVA was performed on the 25 subjects that contributed enough data in both speech and song sessions. There was no significant difference between modalities in the left-frontal ERP familiarity effect (speech: *M(SD*)_novelTest_ = −2.28(1.17), *M(SD)*_familiarizedTest_ = −1.15(1.26); song: *M(SD)*_novelTest_ = −0.86(1.20), *M(SD)*_familiarizedTest_ = 0.75(1.28); *F*_familiarity x modality_(1,24) = 0.045, *p* = 0.83, *ηp*^2^ = 0.002), with also no significant interaction between familiarity, modality, and time window (*F*_familiarity x modality x time window_(1,24) = 0.42, *p* = 0.52, *ηp^2^* = 0.017).

When comparing the ERP familiarity effect across modalities for all electrodes using cluster randomization, one cluster was identified (FC2, CP2) in the 250–500 ms time window, which did not survive multiple comparisons correction (cluster *p* = 0.17). An effect at CP2 was also identified in the 600–800 ms time window, again not surviving multiple comparisons correction (cluster *p* = 0.31). When comparing the ERP familiarity effect across modalities on all time points for the left frontal region of interest, no clusters were identified. Thus, no differences in the ERP familiarity effect were identified for song compared to speech in the test phase.

To summarize, in the familiarization phase, we identified a positive ERP familiarity effect over the left-frontal electrodes in both the 250–500 and 600–800 ms time windows (see Figure 3 and Figure 4). This effect did not differ significantly between the song and speech modality (see Figure 3b,c). In the test phase, there was neither an ERP familiarity effect in song nor in speech.

### 3.3. Follow-Up Analyses—Motivation and Methods

The planned analyses did not render all predicted effects, most notably a positive-going instead of a negative-going familiarity response in the familiarization phase and the absence of any group-level effects in the test phase. As reviewed in the Introduction, the polarity of infants’ responses to familiar words has been associated with stimulus difficulty as well as developmental maturity [71,83], and the absence of group-level effects in previous work has been ascribed to large individual variation in development even within narrow age bands [71]. Therefore, we conducted a series of follow-up analyses to explore individual differences and to assess whether the ERP polarity to the target word shifted with target occurrence during the familiarization phase.

The first follow-up analysis considered the familiarization phase in more detail. In order to expand the comparison of the first two (first/second) versus last two (seventh/eighth) target occurrences from the planned analyses, it assessed how the familiarity responses developed across all eight occurrences of the target word therein. Additional follow-up analyses are reported in Supplementary Table S4, asking whether the lack of a group-level effect in the test phase might be due to a mix of positive and negative responders and associating the responder type to the responses across the familiarization phase.

#### 3.3.1. Follow-Up Analysis #1—Development Over Eight Familiarization Occurrences

**Figure 6:**
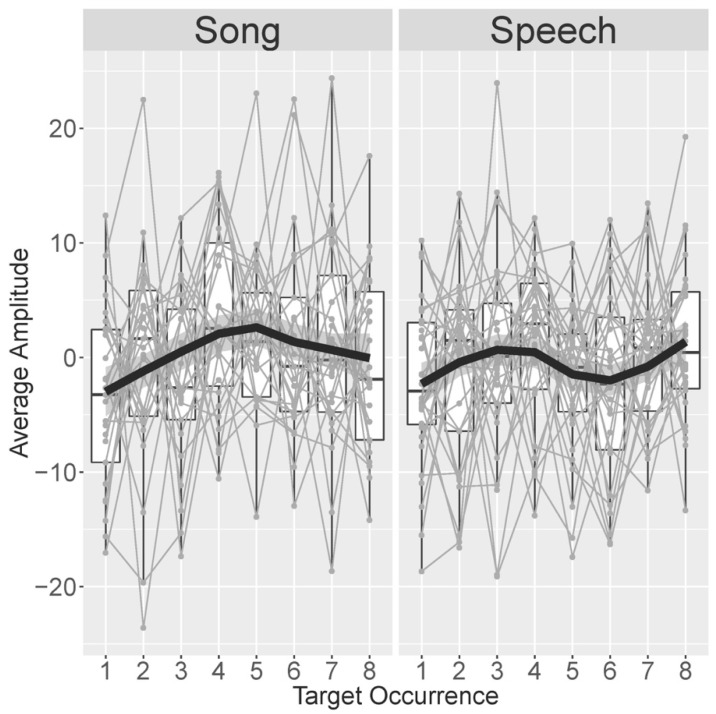
Average electroencephalography (EEG) amplitude in µV (averaged over blocks) in the early (250–500 ms) time window across the eight target occurrences in the familiarization phase in song (left panel) and speech (right panel). Gray lines connect individual participants’ averages, indicated by gray points. The black lines provide a locally estimated scatterplot smoothing (LOESS)-smoothed development averaged over participants.

The first set of follow-up analyses was conducted to scrutinize the identified positive-going response in the last two (seventh/eighth) compared to the first two (first/second) occurrences of the target word in the familiarization passage. Building on the work by Junge and colleagues [89], this analysis targeted the development of the word recognition response across all eight target word occurrences. Figure 6 displays this development, averaged over participants, separated by time window (250–500 ms versus 600–800 ms) and modality (speech versus song). As can be seen in Figure 6 as well as from the model results described below, the word recognition response increased in positivity over the first four occurrences of the target word for both speech and song passages. Then, the response became more negative on occurrences five and six when children listened to speech, but it remained stable when they listened to song. On the final occurrences seven and eight, the word recognition response became more positive again when children listened to speech, whereas it now became more negative when children listened to song.

The statistical analyses were conducted using mixed effects regression models as implemented in the *lmer* function in the *lme4* package [115] in the *R* statistical programming environment [116]. The dependent variable was the per-occurrence average EEG amplitude (in µV) over the left-frontal electrodes (F7, F3, and F5) in the two time windows of interest (250–500 ms and 600–800 ms after stimulus onset), thus adhering to the same electrodes and time windows of interest as in the planned analyses. The random-effects structures were selected to be parsimonious, i.e., containing only those random-effects parameters required to account for the variance in the data, and were determined following an iterative procedure adapted from [117]. The resulting *p*-values were interpreted against the conventional alpha level of 0.05, while *p*-values between 0.05 and 0.07 were interpreted as marginally significant and also worthy of discussion.

The first model assessed the word recognition effect over the eight familiarization occurrences (captured by the first to third-order polynomials, to assess linear, cubic, and quadratic trends over occurrences), comparing across the two modalities (speech = −1; song = 1), the two sessions (first session = −1; second session = 1), and the two time windows (250–500 ms = −1; 600–800 ms = 1). The three fixed factors were fully crossed (i.e., all the possible two-way and three-way interactions were included), and each interacted with the three polynomials. In the first stage of determining the random-effects structure, the dimensionality of the by-subject random effects was reduced to include the (non-correlated) random intercept and slope for modality. In the second stage, the by-word random effects nested within word pairs were added and systematically reduced to by-word random intercepts and by-word slopes for modality and session.

The follow-up models assessed the development of the word recognition effect separately in speech and in song, with as predictors the three polynomials for the eight occurrences, the contrasts for session and time window, the fully crossed fixed effects, and their interactions with the polynomials. To maintain consistency across analyses, we aimed for the random-effects structure of these analyses to contain a by-subject random intercept as well as the by-word random intercepts and slope for session. A model with this random-effects structure converged successfully for the song data, but it had to be reduced to by-subject and by-word random intercepts for the speech data.

The first model, which included both song and speech, revealed a marginally significant positive linear trend over occurrences (β = 51.31, *t* = 1.862, *p* = 0.063), a significant quadratic trend (β = −78.66, *t* = −2.858, *p* = 0.004), and a significant cubic trend (β = 81.74, *t* = 2.968, *p* = 0.003). The linear trend was modulated by a significant positive interaction with session (β = 5.634, *t* = 2.044, *p* = 0.041). The quadratic trend was modulated by a significant interaction with modality (β = −82.44, *t* = −2.996, *p* = 0.003). No other effects were statistically significant or marginally significant. Due to the interactions with session and, especially, modality, the interpretation of these effects requires analyzing subsets of the data. Since the effect of modality was of primary interest, these subset analyses separately analyzed the speech and the song data.

The model on only the speech data revealed a significant cubic trend over occurrences (β = 124.16, *t* = 3.367, *p* = 0.0007) and a significant interaction between session and the linear effect of occurrence (β = 96.52, *t* = 2.619, *p* = 0.009). No other effects were statistically significant or marginally significant. These findings support the observations regarding the speech sessions as made from Figure 6: the EEG amplitude developed non-linearly over the course of a speech block, with two changes in the direction of the developing response (after the initial increase followed a decrease and another increase).

The model on only the song data revealed a significant linear trend over occurrences (β = −94.47, *t* = 2.295, *p* = 0.022) as well as a quadratic trend over occurrences (β = −161.61, *t* = −3.952, *p* < 0.001). There was a marginally significant effect of session (β = −1.393, *t* = −1.904, *p* = 0.065). No other effects were statistically significant or marginally significant. These findings support the observations regarding the song sessions as made from Figure 6: the EEG amplitude developed non-linearly over the course of a song block, with one change in direction of the developing response (the initial increase was followed by a decrease).

These effects were further teased apart in by-occurrence analyses reported in the Supplementary Table S4 (D1: “By-Occurrence analyses”), which confirmed that the difference between modalities was only apparent on occurrences five and six. A final series of models reported in the Supplementary Table S4 (D2: “Occurrence–session interaction”) explored the interaction between the linear trend over occurrences and session, which was apparent in the analyses comparing song and speech as well as the speech-only data. Across the separate analyses of Sessions 1 and 2, the linear effect was only statistically significant in Session 2 of both song and speech data, but it was not statistically significant in Session 1. These results had no implications for the conclusions regarding the development of the familiarity response in speech and song as outlined above.

#### 3.3.2. Additional Follow-up Analyses: Responder Types in the Test Trials

Supplementary Table S4 (D3–5) report additional exploratory follow-up analyses, which suggest that the group-level null effect in the test phase may mask a split between negative and positive responders and thus a robust segmentation effect (D3: “Formally establishing responder types”). Using these groups in further analyses seemed warranted, as there was no strong indication that the positive and negative responders were unequally distributed across the versions (D4: “Experimental control and responder types”). Comparisons of negative and positive responders suggest a more pronounced sinusoid-shaped development across speech familiarization for negative than positive responders (D5: “Comparing development in the familiarization phase across responder types in the test phase”).

## 4. Discussion

The present study set out to test whether infants are able to segment words from ecologically valid children’s songs and then transfer these units to recognition in the spoken register. This was done through an EEG familiarization paradigm for word segmentation [88,89], which presented infants with a series of familiarization-then-test blocks in separate song and speech sessions. Each block commenced with a familiarization phase of one word embedded eight times within an (unique) eight-phrase song or spoken fragment with the same words. The comparison between ERPs to the first two and last two target word occurrences was taken as an index of recognition in the familiarization phase, and speech and song sessions were compared to assess the potential beneficial or hindering effects of song compared to speech. Then, blocks continued with a spoken test phase consisting of two spoken phrases with the familiarized target word and two other spoken phrases containing a matched novel control word to which infants had not been familiarized. The difference in ERP response to the familiar target and control words would index word recognition in the test phase. For the speech sessions, we sought to replicate segmentation from speech as found in the study by Junge and colleagues [89]; for the song sessions, this familiarity effect in the test phase could indicate infants’ ability to transfer words that are segmented from song to recognition in speech.

The planned analyses of the familiarization phase revealed that infants are able to segment words from songs as well as from speech. Specifically, infants’ ERPs showed an increased positivity to the last two compared to the first two target word tokens in both song and speech. This finding shows that infants identify the repetition of word forms within songs with the lyrical and musical complexity of actual children’s songs, thus extending previous results on infants’ abilities to process syllables in segmentally and musically much simpler songs than those employed here [4,5,6]. However, in contrast to this previous work, the present findings provide no clear indication that the segmentation response is stronger in song than speech. Factors that may have contributed to the lack of an observed song benefit are discussed below.

The planned analysis of the test phase provided no evidence of segmentation of either song or speech. This means that the status of the elements that children segment from songs is currently unclear. The interpretation of the absence of song-to-speech transfer is complicated by the lack of evidence for segmentation in the speech test phase following the speech familiarization (thus not replicating [89]). This leaves us unable to provide a definitive answer as to whether infants transfer words that are segmented from song to recognition in speech. Infants’ difficulties with the test phase will be addressed in more detail below.

In addition to these planned analyses, we also conducted a set of follow-up analyses to better understand the unexpected aspects of the results: the positive-going instead of negative-going response in the familiarization phase and the absence of a group-level effect in the test phase. The follow-up analyses of the familiarization data suggested that the response to target words develops non-linearly over a passage and develops differently for song compared to speech. The response to target words in songs showed an inverted U-shape: the positivity increased incrementally over the first four occurrences; then, it remained relatively stable in occurrences five and six and reduced slightly in amplitude in occurrences seven and eight. The response to target words in speech developed with a sinusoid-like shape: regarding song, the positivity increased over the first four occurrences. However, the amplitude then attenuated in occurrences five and six to increase again in the last two occurrences. This was the only difference observed between infants’ responses to song and speech. One could very tentatively consider the inverted U in the development of song responses as a slower version of the first half of the sinusoid development of responses to speech. However, as this analysis was exploratory and the modulation over target occurrences differed from the previously found linear trajectory followed by a plateau at maximum negative amplitude [89], these patterns and thus the potential difference between infants’ processing of speech and songs should be cautiously interpreted until replication.

The exploratory follow-up analyses of the test phase data indicate tentative support for a binary split between infants that displayed a positive versus a negative response to the familiar target versus novel control words. Moreover, infants with a more mature negative response in the test phase showed a stronger modulation over the familiarization phase than the infants with a less mature positive response, which is reminiscent of previous suggestions that infants with negative-going responses have more robust neural segmentation responses across various stages of the procedure [70,93]. While this binary split may explain the absence of a group-level effect in the test phase and the possibility of interpreting the modulation in the familiarization phase, these results await replication, considering the exploratory nature of the binary split between infants with negative-going and positive-going responses (however, see precedents in [69,70,71,93]), as well as the unexpected shape of the modulation of the response over the familiarization phase.

Although the timing and topography of the ERP familiarity effect was comparable to previous reports, the present results differ from previous studies in two critical respects. Firstly, the familiarity response was positive going for both song and speech, instead of the negative-going response that was predicted for the segmentation of these 10-month-old infants [67,79,83,88,92,93]. A positive-going response as observed in the present study is generally considered less mature (see reviews by [71,83]). In the context of speech segmentation, a positivity is associated with segmentation by younger infants [69,83], with infants’ individual poorer concurrent and later language outcomes [67,69,71,93] as well as with stimulus materials that are more difficult to process [69].

A second discrepancy from most of these previous studies is that we found no evidence of segmentation of either speech or song in the test phase. Thus, this (null) finding may qualify claims about the sensitivity of the ERP familiarity response compared to behavioral familiarization-then-test methods [69,80,91,92]. However, the apparent lack of a group-level segmentation response in the present study is in line with the aforementioned recently published results from over 100 9-month-old participants [71] as well as a large-scale failure to replicate speech segmentation in a behavioral task from 8 to 10.5 months [72]. While Kidd and colleagues explain the lack of a group-level response with reference to the large individual differences between infants of roughly the same age and find that the response polarity relates to vocabulary size [71], Floccia and colleagues refer to the acoustic properties of the stimuli [72]. This latter explanation is in keeping with the literature suggesting that the prosodic exaggeration of infant-directed speech facilitates segmentation by infants [85,86]; cf. [87].

The two discrepancies from the literature, a positive-going response and a lack of a segmentation effect in the test phase, could thus be explained with reference to one of two factors: the (linguistic) maturity of the infants, or at least a subgroup of them, and the properties of the stimuli. As we did not collect data about the participants’ concurrent or later language outcomes, the relationship between the EEG responses and (linguistic) maturity cannot be further addressed. However, the role of the stimulus properties can be addressed by directly comparing the acoustic features of the present stimulus set to the stimuli from Junge and colleagues [89], who found a negative-going response in the familiarization as well as the test phase. For a valid comparison between the present stimuli and those of [89], we re-analyzed the average pitch and pitch range of the stimuli of Junge and colleagues and added the acoustic focus measure, using the same procedure as reported in the present Methods section. The duration values to quantify speaking rate are taken directly from [89].

This comparison between the stimulus sets will focus on three features that have been suggested as facilitating infant speech processing in the literature. First, an overall higher pitch with more expanded range is often cited as the prime difference between IDS and adult-directed speech (ADS) (for reviews: [7,118], facilitating segmentation on the basis of transitional probabilities [85,86] as well as possibly segmentation from natural speech [72]; c.f., [87]. However, exaggerated pitch properties have not been found to enhance infant word recognition [119]. Second, pitch changes may also provide a specific acoustic focus on the target word. This focus appears essential for segmentation by 6-month-olds, and still aids segmentation by 9-month-olds [83]. Third, a slower speaking rate is often cited as a key difference between IDS and ADS (cf. [120]), which benefits infants’ word-processing efficiency [119]. A fast speaking rate is disruptive to infants’ segmentation abilities, eliminating a group-level response for 11-month-olds and shifting the behavioral response to a less mature familiarity preference in 14-month-olds [121].

Of the three features scrutinized, overall, pitch characteristics probably do not affect the maturity of the speech segmentation response observed in the present study: The average pitch was in fact higher in the present speech stimuli compared to those used by [89] (234 Hz versus 201 Hz), and the pitch range was highly comparable between the two datasets (10.90 semitones versus 10.28 semitones). Unless a lower-pitched voice is easier to segment, the present data support the conclusion from [119] that overall, pitch characteristics probably do not facilitate infant word processing, extending the conclusion to segmentation. The specific acoustic emphasis on the word could be a contributing factor to the relatively immature speech segmentation response in the present study, as the acoustic focus on the target word was somewhat smaller in the present compared to the [89] stimuli (0.896 versus 0.930, respectively). This suggests that prosodic focus may still aid segmentation at 10 months, extending the age range for which prosodic focus is found to be beneficial from 9 to 10 months [83]. Finally, the speaking rate of the stimuli could (partially) account for the positive-going response. The speaking rate in our speech stimuli is faster than in [89], as evidenced by shorter target words and phrases (target words: 515 ms versus 694 ms, respectively; phrases: 2068 ms versus 2719, respectively) despite both studies using disyllabic trochees as target words and having a very similar number of words per phrase (5.71 versus 5.75). This comparison agrees with the possible beneficial effect of a slow speaking rate for word recognition [119] and the hindering effects of a fast speaking rate for word segmentation [121].

The suggestion that acoustic prominence and speaking rate facilitate segmentation is also tentatively supported by the properties of the test phase stimuli. Recall that no group-level segmentation effect was observed in the test phase, but that about half of the participants displayed a (mature) negative-going response. Interestingly, the test stimuli were spoken with more acoustic focus on the target words than the familiarization stimuli (0.942 versus 0.896, respectively), which is comparable to the focus in the stimuli from Junge and colleagues ([89]; 0.930). Moreover, the test stimuli were somewhat slower than the familiarization stimuli, although not as slow as the [89] stimuli (target word duration: 538 ms; phrase duration: 2216 ms). In other words, the test stimuli might have been somewhat easier to segment than the familiarization stimuli. This might have facilitated a negative-going response in a larger subset of participants (or trials), possibly resulting in the disappearance of the group-level positive-going effect from the familiarization phase.

The present stimuli may have emphasized the target words occurring in the familiarization phase less than previous studies, because our stimuli were recorded in short stories rather than in individual sentences. Target words may be emphasized less in narratives, as the emphasis on repeated words diminishes over subsequent utterances, even in infant-directed speech [122]. If the recording of narratives and associated reduced prosodic emphasis is indeed responsible for the present study’s less mature segmentation response, this raises questions about infants’ ability to segment words from the speech that they are presented with during their daily interactions.

However, the acoustic properties of the stimuli do not explain all aspects of the present data, which becomes apparent when the song stimuli are considered. The song stimuli provided more prosodic focus on the target words (0.959) and were lower in speaking rate (target word duration: 794; phrase duration: 3181 ms), even compared to the stimuli of Junge and colleagues [89]. If acoustic focus and/or a slow speaking rate were necessary and sufficient for a mature segmentation response, the song familiarization should have elicited a group-level negative-going response or enabled more children to display this response. Thus, the observed positivity in the song familiarization could reflect additional challenges segmenting words from songs, such as the atypical acoustic signal of sung compared to spoken language [41,42]; cf. [43], or the increased risk of mis-segmentations from songs [44,45,123]. Alternatively, the acoustic focus and speaking rate might not explain the positive-going response in the present speech familiarization either, in which case we need to look to other factors to explain the different result patterns across studies.

One possible deviation in procedure compared to some previous studies (e.g., [88,89]) is that to maintain experimental engagement, besides showing desynchronized visual stimuli on a computer screen, in the present study, not only the parent but also an experimenter sat with the infant and showed silent toys when necessary. Note that other experimenters (e.g., [71], who did not find a group level effect in 9-month-olds) have also used such an entertainer. The additional visual distraction might have resulted in less selective attention to the auditory stimuli for at least some of the children or trials. Note that the allocation and maintenance of attentional focus undergo major developmental changes in infancy, with individual differences herein possibly being related to lexical development [124]. The precise effect of visual distraction (life or on screen) on the ERP word familiarity effect should be established in future studies. If life visual distraction were found to reduce or eliminate a mature negative-going response at the group level, this would raise questions about the effect of selective attention to auditory stimuli on the ERP familiarity effect in general. Is a negative ERP word familiarity effect possibly a reflection of more focused attention to the auditory stimuli, and do children who are better at selectively attending to auditory stimuli then develop language quicker (as in [71])?

As described in the Introduction, the developmental shift from an initial positivity to a later negativity for word recognition responses has been ascribed to cortical maturation as well as to the development of the lexicon [71,83], with various stimulus and task characteristics giving rise to a ‘lexical processing mode’, resulting in the negative ERP familiarity effect. This repetition enhancement effect might reflect the strengthening of novel neural representations during active learning ([103]; see Introduction). The positive ERP familiarity effect for both speech and song stimuli for the 10-month-olds in the current study suggests that acoustic prominence, speaking rate, and selective auditory attention might be important to get infants into such an active lexical processing mode that is necessary for building a lexicon. Future research is needed to further clarify the development and precise characteristics of the infant ERP familiarity effect and to specify the exact role of lexical processing in its polarity—for example, by actively manipulating the lexical status of stimuli or the processing mode of the infant.

The absence of evidence for better segmentation from song compared to speech, although not the primary focus of the present study, could be considered surprising in light of the previous research finding benefits of songs for language learning in adults and infants [4,5,6]. Two factors might have contributed to the lack of a song advantage in the present study. First, the present speech stimuli might have been more attractive than those used in the previous infant studies comparing language learning from song and speech. The speech stimuli of previous work were spoken in a completely flat contour [6], adult-directed speech [4], or at a pitch intermediate between infant-directed and adult-directed speech [5]. In contrast, the present stimuli were elicited in an infant-directed register and had an overall pitch that was highly comparable to the average pitch in a sample of Dutch-speaking mothers interacting with their own 15-month-old infant (236/237 Hz and 234 Hz, respectively; [125]). Thus, the lack of a song advantage in the present study could reflect the beneficial effects of infant-directed speech for infant speech segmentation [85,86]; cf. [87]. A second factor contributing to the lack of an observed song advantage might have been the relative complexity of the songs in the present study. The stimuli of previous work all presented consistent syllable–tone pairings, which were combined into one [5], two [4], or four [6] four-syllable melodies that were repeatedly presented to the infants during familiarization. In contrast, the present stimuli consisted of 20 novel melodies, some of which included a melodic repetition in the second four-phrase verse. As melodic repetition facilitates word recall from songs [24], the children’s songs in the present study may have been too novel and complex to enhance segmentation. We are looking forward to future research investigating whether song complexity and, in particular, experience with the songs increases the benefit of songs for word learning.

In sum, this study provided electrophysiological evidence that 10-month-old Dutch children can segment words from songs as well as from speech, although the segmentation response was less mature than that observed in previous studies and did not persist into the test phase. Close inspection of the stimuli suggested that a limited prosodic focus on the target words and a relatively fast speaking rate may have inhibited more mature responses to the speech stimuli. However, these same cues were strongly present in the song stimuli, suggesting that other factors may suppress mature segmentation from songs. Future research will need to establish which aspects of songs, including children’s familiarity with them, contribute to children’s segmentation from speech and song and to what extent children can recognize words segmented from songs in speech.

## Figures and Tables

**Table 1:** Example of a familiarization +test block with target word pair bellers-piefen. Target words are underlined. Materials were in Dutch. English word-for-word and semantic translations are given. The first two (first/second) target occurrences are indicated in blue, the last two (seventh/eighth) target occurrences of the familiarization phase are indicated in red. Familiarized target words in the test phase are indicated in purple, and novel control words are indicated in green.

Familiarization Phase (Sung or Spoken)		Word-for-Word Translation	Semantic Translation
	Luister eens!	Listen once!	Listen to this!
1.	Met bellers kun je lachen	With callers can you laugh	One can have a laugh with callers
2.	De vrouw vindt bellers stom	The woman regards callers stupid	The woman thinks callers are stupid
3.	We spraken met de woeste bellers	We spoke to the wild callers	We spoke to the wild callers
4.	Dan praten bellers graag	Then speak callers preferably	Callers prefer to speak then
5.	Daar achterin zijn bellers	There behind are callers	Callers are there in the back
6.	Wat lopen bellers snel	What walk callers fast	Callers walk that fast
7.	Jouw bellers kletsen makkelijk	Your callers chat easily	Your callers are at ease chatting
8.	Ik zag de bellers niet	I saw the callers not	I did not see the callers
**Test phase (spoken)**			
	Luister eens!	Listen once!	Listen to this!
9.	Aan die piefen gaf hij koffie	To those hotshots gave he coffee	He served those hotshots coffee
10.	Vaak gaan bellers op reis	Often go callers to travel	Callers travel often
11.	Alle bellers stappen laat uit	All callers get late off	All callers get off late
12.	Zij zijn goede piefen geworden	They have good hotshots become	They have become good hotshots

**Table 2:** The 20 target word pairs (English translation in parentheses), and songs that were the base for the melody of that specific word pair (language of lyrics of original song in parentheses).

	Word 1		Word 2		Song that Melody Was Based on
1	bellers	(callers)	piefen	(hotshots)	If all the world were paper (English)
2	hinde	(doe)	emoe	(emu)	Sing a song of sixpence (English)
3	gondels	(gondolas)	schuiten	(barques)	See-saw Margery Daw (English)
4	drummer	(drummer)	cantor	(cantor)	Georgie Porgie (English)
5	gieter	(watering cans)	silo’s	(silos)	There was a crooked man (English)
6	hommels	(bumblebees)	kevers	(beetle)	Pat-a-cake (English)
7	fakirs	(fakirs)	dansers	(dancers)	Little Tommy Tucker (English)
8	krekels	(crickets)	hoenders	(fowl)	En elefant kom marsjerende (Norwegian)
9	krokus	(crocus)	anjer	(carnation)	Smil og vær glad (Norwegian)
10	lener	(borrower)	preses	(president)	Ute På Den Grønne Eng (Norwegian)
11	mammoet	(mammoth)	orka	(orca)	Jeg snører min sekk (Norwegian)
12	monnik	(monk)	frater	(friar)	Auf de Swäb’sche Eisenbahne (German)
13	otters	(otter)	lama’s	(llamas)	Suse, liebe Suse (German)
14	mosterd	(mustard)	soja	(soya)	Schneeflöckchen Weißröckchen (German)
15	pelgrims	(pilgrim)	lopers	(runners)	Wem Gott will rechte Gunst erweisen (Ger.)
16	pudding	(pudding)	sorbet	(sorbet)	Wiesje (Dutch)
17	ronde	(round)	kuier	(saunter)	A l’intérieur d’une citrouille (French)
18	sitar	(sitar)	banjo	(banjo)	La bonne avonture o gué (French)
19	sultan	(sultan)	viking	(Viking)	Neige neige blanche (French)
20	zwaluw	(swallow)	kievit	(lapwing)	Entre le boeuf e l’âne gris (French)

## Supplementary Materials

The following are available online at https://www.mdpi.com/2076-3425/10/1/39/s1, Table S1: Acoustic Properties of the Experimental Stimuli, Table S2: Pre-Test on the Melodies, Table S3: Full Set of Stimulus Materials, Table S4: Additional Analyses to the Follow-up Analyses.
