# Supplementary material for: Infants Segment Words from Songs—An EEG Study
Source: Brain Sci. 2020 Jan 9;10(1):39. doi: 10.3390/brainsci10010039 (PMC7017257; doi:10.3390/brainsci10010039)
Supplement: Supplementary file 1 [file brainsci-10-00039-s001.pdf]

Supplementary Materials

A. **Acoustic Properties of the Experimental Stimuli** **p. 2**

B. **Pre-test on the Melodies** **p. 4**

C. **Full Set of Stimulus Materials** **p. 7**

D. **Additional Analyses to the Follow-up Analyses** **p. 11**

Supplementary Materials A - Acoustic Properties of the Experimental Stimuli

Distracting noises were removed from the stimuli by setting the sound to silent or by removing the noise from the signal, always cutting at zero crossings. The loudness in all test sentences and attentional phrases was scaled to 70 dB, as was the average loudness over all phrases in a familiarization recording (thus, excluding the intervals between the phrases). Duration, pitch, and loudness measures are given in Supplementary Table S1 (for phrases) and Supplementary Table S2 (for target words). The mean 'focus' is also reported in Supplementary Table S2, which is the maximum F0/intensity in the target word divided by the maximum F0/intensity in the phrase. The 'focus' measure approaches 1 if the target word is always the highest or loudest, and thus most prominent, in the phrase. To prevent F0 measurement errors from affecting the focus measure, the 90th percentile of the F0 in the phrase was considered as its maximum for these computations. All F0 tracks were hand-corrected prior to the measurements, to prevent pitch halving and doubling errors.

We made sure the first two (first/second) and last two (seventh/eighth) occurrences of the target words were matched on duration, F0, and F0 range, and the degree of F0 change compared to the previous word (see Supplementary Table S3).

Table S1. Acoustic properties of the phrases in the familiarization and test stimuli. Averages (and sd) are computed over all tokens from all stimuli. Interval duration gives the time in between phrases.

	phrase duration (ms)	phrase median F0 (Hz)	phrase F0 range (St)	phrase median intensity (dB)	interval duration (ms)
Song - familiarization	3,181	332	6.89	67.25	492
	(869)	*(52)*	*(2.34)*	*(2.40)*	*(322)*
Speech - familiarization	2,068	237	10.90	66.89	954
	(447)	*(30)*	*(2.12)*	*(1.96)*	*(324)*
Speech - test	2,216	236	10.65	67.00	n/a
	(269)	*(27)*	*(2.00)*	*(1.41)*	*n/a*

Table S2. Acoustic properties of the target words in the familiarization and test stimuli. Averages (and sd) are computed over all tokens from all stimuli. The "focus" measures provide maximum F0/intensity in the target word divided by the maximum F0/intensity in the phrase.

	Duration (ms)	F0(Hz)		Intensity (dB)	
	word	word	focus (pitch)	word	focus (loudness)
Song - familiarization	*794*	337	0.959	68.09	0.967
	(222)	*(62)*	*(0.109)*	*(3.29)*	*(0.032)*
Speech - familiarization	*515*	252	0.896	67.60	0.966
	(108)	*(55)*	*(0.165)*	*(3.08)*	*(0.034)*
Speech - test	*538*	*261*	0.942	67.32	0.962
	(80)	*(41)*	*(0.112)*	*(3.00)*	*(0.032)*

Table S3. Acoustic properties of the target words in phrases 1 & 2 versus those in phrases 7 & 8 of the familiarization stimuli. Averages (and sd) are computed over all tokens in these phrases of the familiarization stimuli. The reported *t*- and *p*-values are those of paired-samples *t*-tests, comparing the 80 values taken from phrases 1 and 2 in each of the 40 familiarization stimuli to the matched values from phrases 7 and 8. These t-values are therefore conducted with df=79.

		Word 1/2	Word 7/8	*t*	*p*
Duration (in ms)	Song	792 (*171*)	754 (*178*)	1.383	0.171
	Speech	507 (*96*)	491 (*86*)	1.605	0.112
Pitch (in Hz)	Song	331 (*58*)	345 (*66*)	-1.511	0.135
	Speech	267 (*60*)	259 (*50*)	1.013	0.314
Pitch Range (in St) (90th percentile minus 10th percentile)	Song	2.74 (*1.54*)	2.41 (*1.61*)	1.265	0.209
	Speech	5.16 (*3.13*)	5.26 (*3.15*)	-0.201	0.841
Pitch change from preceding word (median Hz in target word minus preceding word)	Song	-2 (*50*)	5 (*71*)	-0.640	0.524
	Speech	13 (*89*)	-9 (*76*)	1.846	0.069

Supplementary Materials B– Pre-test on the Melodies

This section of the supplementary materials describes the procedure followed to select melodies for the stimulus materials that are unfamiliar to 10-month-old Dutch infants.

Participants

Twenty-two parents of 10-month-old infants completed the pre-test questionnaire. Participants were all recruited from the database of the Baby and Child Research Center. Parents who participated were rewarded with a small children's book that was sent to them via mail. None of the parents that were contacted for the pre-test participated with their infant in the actual experiment.

Stimuli

An initial set of 29 melodies of children's songs consisting of either eight or four phrases was found through a search on the internet and in printed book collections. These melodies were originally German (n=5), English (n=9), French (n=8), and Norwegian (n=7) and unfamiliar to Dutch infants as judged by the three authors and a fourth collaborator (all native speakers of Dutch). An additional four familiar Dutch children's songs and four unfamiliar Dutch children's songs (familiarity judged by the same four people) were selected to serve as the manipulation check. These 29 non-Dutch and 8 Dutch melodies, or slight variations thereof, were recorded in a sound-attenuated studio by a musically trained assistant, who replaced the words by singing "la" to each individual note.

Procedure

The 37 recorded melodies were divided over four lists. Each melody occurred on two lists, and each list contained two or three German, four or five English, four French, three or four Norwegian, two familiar Dutch and two unfamiliar Dutch melodies. The melodies in each list were presented in a fixed order, with the presentation order meeting the restrictions that 1) no two melodies from the same language were presented subsequently; 2) approximately the same number of songs from each language was presented in the first and second half of each list; 3) one familiar and one unfamiliar Dutch melody were presented in the first half of the list and the other two Dutch melodies in the second half of the list; and 4) a melody that was presented in the first half of one list was presented in the second half of the other list.

Four versions of the pre-test, corresponding to the four lists, were implemented in an on-line experiment using the software program Net-Questionnaire. Parents who agreed to participate received an e-mail with instructions and a link to the version of the pre-test that was assigned to them (ranging from four to seven participants per version). Participants first filled out basic questions about their parenting experience. They were then presented with 18 or 19 of the recorded melodies and asked to answer the seven questions outlined in Table S4 for each melody. The total duration of the pre-test was approximately 15 minutes.

Results

The average familiarity ratings of the four unfamiliar Dutch children's songs were 3.18 for the melody (question 1) and 2.05 for the lyrics (question 2) and more than a full point below the average familiarity ratings for the four familiar Dutch children's songs (4.85 for the melodies and 4.49 for the lyrics). This indicates that the questions on familiarity successfully captured this key aspect of parents' impression of the songs.

Table S4. Questions (English translations from Dutch originals) asking about participants' familiarity with and impression of each melody in the pre-test.

	Question	Answer type	Answer detail
1	To what extent is this melody familiar to you?	5-point likert scale	"1" indicates: Not at all, I hear this melody for the first time.<br>"5" indicates: Absolutely, I could have hummed along immediately
2	To what extent do you know the lyrics that go with this melody?	5-point likert scale.	"1" indicates: Not at all<br>"5" indicates: Very well, I could sing the text without hesitation
2b	If you know the text to some extent, please provide the title or some of the lyrics.	Optional free-text response	
3	Do you think that your (youngest) child is familiar with this melody?	Yes/No question	
3b	If your child knows the melody, does (s)he know it from…	Multiple choice question, multiple answers possible	Response options:<br>singing at daycare;<br>a cd;<br>television or internet;<br>other (with the free-text option to provide an explanation).
4	Could this melody be or become a Dutch children's song (as well)?	5-point likert scale.	"1" indicates: No, not at all, this melody does not at all sound like Dutch children's songs.<br>"5" indicates: Yes, absolutely, this melody could be or become a Dutch children's song (as well).
5	To what extent do you think that children will like this melody?	5-point likert scale.	"1" indicates: Not at all, I think they would not want to listen to this melody.<br>"5" indicates: Very much, I think they would listen attentively to this melody.

Melodies were considered suitable for the experiment if parents consistently rated the melody as well as the text as unfamiliar to themselves (questions 1 and 2). Melodies were prioritized if parents rated them as suitable for a Dutch children's song and as relatively attractive to children (questions 4 and 5). Parents' rating of the familiarity of the melody to the child was not taken into account, as the parents often rated the Dutch songs as less familiar to their child than the non-Dutch songs, despite the former being more familiar to the parents than the latter. This suggested that parents do not report with confidence that their child is unfamiliar with songs that they themselves are unfamiliar with, rendering the child's familiarity with a song an unreliable selection criterion for our purposes. On the basis of these criteria, 20 songs were selected for the experiment, including one of the unfamiliar Dutch children' songs. Table S5 provides an overview of the mean ratings of the selected songs.

Table S5. The songs whose melodies were included in the stimuli for the experiment and the average ratings received on those questions from the pre-test that served as selection criteria. Q1=Familiarity of the Melody; Q2=Familiarity of the Lyrics; Q4=Suitability of the melody for a Dutch children's song; Q5=Attractiveness of the melody for children. Refer to Table S4 for the exact wording of the questions.

Original Song Title	Original Language	Q1	Q2	Q4	Q5
If all the world were paper	English	1.45	1.00	4.36	4.27
Sing a song of sixpence	English	1.36	1.00	3.73	3.45
See-saw Margery Daw	English	1.64	1.09	3.36	3.45
Georgie Porgie	English	1.55	1.27	3.09	3.00
There was a crooked man	English	1.00	1.00	3.67	4.11
Pat-a-cake	English	1.00	1.00	3.44	2.78
Little Tommy Tucker	English	1.22	1.00	3.44	3.67
En elefant kom marsjerende	Norwegian	1.54	1.08	3.38	3.38
Smil og vær glad	Norwegian	1.09	1.00	2.73	2.82
Ute På Den Grønne Eng	Norwegian	1.67	1.00	3.56	3.78
Jeg snører min sekk	Norwegian	1.11	1.00	3.33	3.44
Auf de Swäb'sche Eisenbahne	German	1.77	1.08	3.69	3.15
Suse, liebe Suse	German	1.91	1.09	3.55	3.27
Schneeflöckchen Weißröckchen	German	1.18	1.00	3.18	2.64
Wem Gott will rechte Gunst erweisen	German	1.22	1.00	3.44	3.78
Wiesje	Dutch	1.73	1.09	3.73	3.00
A l'intérieur d'une citrouille	French	2.00	1.00	3.91	4.09
La bonne avonture o gué	French	1.55	1.00	3.73	3.73
Neige neige blanche	French	1.00	1.00	3.56	3.89
Entre le boeuf e l'âne gris	French	1.67	1.11	2.78	2.33

Supplementary Materials C - Full Set of Stimulus Materials (text)
The actual sung and spoken stimuli will be made available on OSF.

Target words	Familiarization phrases	Test phrases
1. bellers/piefen	Met bellers/piefen kun je lachen	Aan die piefen gaf hij koffie
	De vrouw vindt bellers/piefen stom	Vaak gaan bellers op reis
	We spraken met de woeste bellers/piefen	Alle bellers stappen laat uit
	Dan praten bellers/piefen graag	Zij zijn goede piefen geworden
	Daar achterin zijn bellers/piefen	
	Wat lopen bellers/piefen snel	
	Jouw bellers/piefen kletsen makkelijk	
	Ik zag de bellers/piefen niet	
2. hinde/emoe	‘k Wil een hinde/emoe vangen	Mooie emoe komt uit Australië
	‘s Nachts gaat hinde/emoe weg	Vrolijk kijkt één hinde ons aan
	‘t Hertje houdt van hinde/emoe	Een aardige hinde weet de weg
	‘k Zag zo'n hinde/emoe graag	Daar loopt emoe van de boerderij
	Wij vangen jouw hinde/emoe	
	Geen hinde/emoe op het spoor	
	Naast die hinde/emoe loopt een geit	
	Wat is de hinde/emoe zwaar	
3. gondels/schuiten	Al die gondels/schuiten zijn goed	Deze schuiten zien er groot uit
	Onder de gondels/schuiten zijn vissen	Alle gondels varen snel weg
	En houten gondels/schuiten die vindt hij zo eng	Daar gaan gondels vaak heen
	Ze voeren hier langs in hun gondels/schuiten	De koning heeft schuiten genoeg
	Erg ruim zijn gondels/schuiten niet	
	Het lijken wel blauwe gondels/schuiten	
	Nieuwe gondels/schuiten zijn hier toch allang	
	Vandaag komen gondels/schuiten uit Spanje	
4. drummer/cantor	Hij was drummer/cantor van een orkest	Zo'n cantor haalt alle noten
	Voor je drummer/cantor stond het klaar	Meteen sloeg mooie drummer op hol
	Van de drummer/cantor was de trom	Op slag was haar drummer verliefd
	Want ik vind een drummer/cantor gek	Ik zag hun cantor op televisie
	‘t Bandje is zijn drummer/cantor net kwijt	
	Deze drummer/cantor die zong graag	
	Onze drummer/cantor was zo zoet	
	En die drummer/cantor maakt muziek	
5. gieters/silo's	De boer heeft gieters/silo's vast	Zulke silo's zijn alleen voor het land
	Op het plein staan gieters/silo's klaar	Op de weg zie je gieters nooit
	Geen tuin kan zonder gieters/silo's	Achterin zijn gieters verstopt
	De meid ziet gieters/silo's graag	Hij wil silo's voor dat geld
	De plant vindt gieters/silo's fijn	
	Maar gieters/silo's waren op	
	Door de gieters/silo's stroomt water	
	En twee gieters/silo's staan al daar	
6. hommels/kevers	Goede zin krijg je met hommels/kevers niet	Grote kevers vliegen in het licht
	Steeds meer hommels/kevers zien we hier gaan	Dat is voor hommels erg prettig
	Ik zag daar hommels/kevers in zwermen door de lucht	De hommels zijn heel erg klein
	Flamboyante hommels/kevers zijn bloemengek	De oude kevers zijn zeer zeldzaam
	Egel zwaait naar de drie hommels/kevers daar	

	In de lucht vliegen hommels/kevers voorbij<br>In de stad zijn die hommels/kevers blij en heel uniek<br>Volgens haar hommels/kevers heb ik nu pech	
7. fakirs/dansers	Dove fakirs/dansers zien we<br>Oude fakirs/dansers niet echt<br>Daar liggen fakirs/dansers<br>Messen voor de fakirs/dansers<br>Maken fakirs/dansers circus<br>Zij willen fakirs /dansers zien<br>Zouden fakirs/dansers snappen<br>Dat fakirs/dansers mager zijn	Die enge dansers zijn magisch<br>Een leeuw maakt deze fakirs bang<br>Weer zijn de mooie fakirs te laat<br>Alle dansers doen hun best
8. krekels/hoenders	Ik zag vele krekels/hoenders in het gras<br>Weinig krekels/hoenders maken veel lawaai<br>Rond de boom staan krekels/hoenders vaak rechtop<br>In de wouden waren groene krekels/hoenders overal<br>Prinsen zien vaak krekels/hoenders grinniken<br>In de sprookjes spreken krekels/hoenders goed<br>Want ik zag de krekel/hoenders s op het feest<br>Avonturen met die krekels/hoenders pakken wel goed uit	Met zulke hoenders kun je lachen<br>Vier vrolijke krekels zijn er al<br>Van drop houden krekels veel<br>Die grote hoenders zijn eng
9. krokus/anjer	Naast kleine krokus/anjer liep grote mier<br>Ondanks de sneeuw kwam een krokus/anjer op<br>In het groene gras stak de krokus/anjer fier omhoog<br>Daar bij de paarse krokus/anjer is het mos wat weggehaald<br>Ik zag de bloeiende krokus/anjer staan<br>Lang stond mijn krokus/anjer alleen in 't bos<br>Wist je dat ik die krokus/anjer gister heb geplukt<br>De roze krokus/anjer vond het blije meisje 't allermooist	Zo'n anjer zie je in sommige landen heel veel<br>Net naast krokus ligt wat troep<br>De mooiste soort krokus is oud<br>Deze anjer is erg opvallend
10. lener/preses	Dat betaalt mijn lener/preses wel<br>Daar springt lener/preses ver vooruit<br>Vader zag de lener/preses daar<br>Snelle lener/preses loopt gauw weg<br>Crisis met hun lener/preses stopt<br>Dapp're lener/preses viert een feest<br>Veel krijgt kleine lener/preses niet<br>Gister viel een lener/preses af	De erg strenge preses geeft op<br>Aan haar lener lag het niet<br>Elke lener krijgt vier boeken<br>De nare preses gaat weg
11. mammoet/orka	In vroegere tijd, lag mammoet/orka op het ijs<br>En spreken dat kon mijn mammoet/orka zeker nog niet<br>De mammoet/orka van toen, die vierde graag een feest<br>De jonge mammoet/orka die gaat graag jagen<br>Vier eeuwen terug, stierf onze mammoet/orka uit<br>En onderwijl past jouw mammoet/orka overal bij<br>Maar stortregen maakt haar mammoet/orka steeds weer nat<br>Het jaarboek heeft geen mammoet/orka als wapen	Ik zag die orka op de televisie<br>Niet één mammoet bleef thuis<br>Straks gaat mammoet op reis<br>Deze orka kan heel goed kunstjes leren
12. monnik/frater	Elke dag bidt monnik/frater uren<br>Ieder vroeg zijn monnik/frater raadsels<br>Deze monnik/frater staat in 't licht<br>Aan de monnik/frater is niets te zien<br>Hij was een heel leuke monnik/frater<br>Niets is te veel voor die monnik/frater	De koning hoort de boze frater vallen<br>De mug wil geen monnik spreken<br>De tuin van jouw monnik is netjes<br>Straks maakt frater weer rare grappen

	Volgens monnik/frater komt het goed<br>Strenge monnik/frater draagt een kap	
13. otters/lama's	Witte otters/lama's zwemmen<br>Twee otters/lama's te voet<br>En nu vlug, daar gaan de otters/lama's<br>Want otters/lama's staan graag<br>De jongen houdt otters/lama's<br>Meer otters/lama's dan ik<br>En de man, die hield drie otters/lama's vast<br>Die otters/lama's daar	Dat zijn lama's uit de dierentuin<br>Jouw otters zijn niet zo bekend<br>Van slapen hielden otters het meest<br>Verbaasd liepen er lama's weg
14. mosterd/soja	Zijn mosterd/soja is zurig<br>Worst met mosterd/soja is goed<br>Hij houdt echt niet van die mosterd/soja<br>Hij maakt mosterd/soja liefst zelf<br>Zij zocht naar de mosterd/soja<br>Noorse mosterd/soja is top<br>‘t Meisje wil geen mosterd/soja eten<br>Nieuwe mosterd/soja vandaag	Toch is deze soja al lang zeldzaam<br>Hij spaart mosterd uit Frankrijk<br>Grove mosterd staat op tafel<br>Men vindt soja vaak niet lekker
15. pelgrim/lopers	De oude pelgrims/lopers slapen dieper<br>De Ierse pelgrims/lopers rennen weg<br>Fietsen is een hobby van de pelgrim/lopers<br>Want altijd komen pelgrims/lopers/lopers langs<br>Het klooster zoekt nog steeds twee pelgrims/lopers<br>Dat komt die pelgrims/lopers wel goed uit<br>Deze pelgrims/lopers groeten de boeren<br>Want haar pelgrims/lopers zijn al weg	Alle lopers wisten alles<br>Van wachten houden pelgrims ook<br>Ze ziet grillige pelgrims liggen<br>Hij zwaait nu zijn lopers uit
16. pudding/sorbet	Na de pudding/sorbet drink ik graag warme melk<br>Juf wil pudding/sorbet toe<br>De koelkast geeft zijn pudding/sorbet kou<br>Er zal echt pudding/sorbet zijn<br>Bovenaan zijn lijst staat een pudding/sorbet, want<br>Zoete pudding/sorbet maak je snel<br>De lekk’re pudding/sorbet smaakt heel fris<br>De beste pudding/sorbet heeft fruit	Een warme sorbet is niet lekker<br>Ze krijgt pudding na het eten<br>Gele pudding is koud beter<br>Die sorbet is heel smaakvol
17. ronde/kuier	De krokodil is een ronde/kuier verder<br>Ik zag de lange ronde/kuier staan<br>Dagelijks loopt bij braaf zijn ronde/kuier<br>En zo ziet u mijn ronde/kuier graag<br>Deze ronde/kuier, was een echte klus<br>‘s Nachts loop ik een betere ronde/kuier<br>Voor de vrouw is de ronde/kuier lang<br>Na elke ronde/kuier namen zij rust	Het lijkt een goede ronde te zijn<br>De laatste kuier wint de rups<br>De bloemist is geen kuier verder<br>Zo'n ronde is goed te doen
18. sitar/banjo	Nu zie je mijn sitar/banjo niet<br>Daar kun je hun sitar/banjo zien<br>Zo speelt de sitar/banjo hier zijn spel<br>‘k Denk da'k deze sitar/banjo koop<br>Popster pakt zijn sitar/banjo uit<br>Onze sitar/banjo is gestemd<br>Met je sitar/banjo maak je klank	Wat is de banjo toch mooi<br>Een sitar zie je soms op straat<br>Elke sitar werd verkocht<br>De dienaar legt haar banjo weg

	Op jouw sitar/banjo zit een snaar	
19. sultan/viking	‘k Zag zijn sultan/viking rijden<br>Reist een zware sultan/viking<br>Voor haar sultan/viking staat een maal<br>Zo'n sultan/viking helpt zijn slaaf<br>Zie die strenge sultan/viking<br>Je sultan/viking is heel machtig<br>Ga gerust met sultan/viking uit<br>Want de sultan/viking lacht	Die kleine viking is niet sterk maar slim<br>Ijverig zoekt jouw sultan zijn kat<br>Een belangrijke sultan heeft macht<br>Daarmee maakt viking veel vijanden
20. zwaluw/kieviet	De zwarte zwaluw/kieviet zoeft voorbij<br>Boven zie je zwaluw/kieviet gaan<br>Hij kijkt naar de kleine zwaluw/kieviet die daar vliegt<br>Zijn vrouw wacht nog op haar zwaluw/kieviet daarginds<br>Gister vloog echt de zwaluw/kieviet weg<br>Naast mijn zwaluw/kieviet vliegt een bij<br>Een gestreepte zwaluw/kieviet is heel zeldzaam hier<br>Vroeg in de zomer is zwaluw/kieviet hier al	De andere kieviet zie je nooit<br>Er was één zwaluw bij de sluis<br>Men ziet geen zwaluw in het bos<br>Die kieviet zit in een hoge boom

Supplementary Materials D – Additional analyses to the Follow-up analyses

D1. By-occurrence analyses (supplement to Follow-up analysis #1 – development over eight familiarization occurrences)

The differential development of the word recognition response between speech and song was further confirmed by a series of analyses comparing the speech and song data across pairs of occurrences (occurrence 1 versus 2; 3 versus 4; etc.). These analyses included the linear contrast between the occurrences (e.g., occurrence 1=-1, occurrence 2=1) and further retained the fixed effects structures from the main analysis, namely comparing across the two modalities (speech=-1; song=1), the two sessions (first session=-1; second session=1), and the two time windows (250-500 ms=-1; 600-800 ms=1).

We aimed for the same random-effects structure as used in the first model addressing the development over familiarization occurrences (i.e., a by-subject random intercept and slope for modality as well as by-word random intercepts and slopes for modality and session). Due to convergence issues in the model for occurrences 5 and 6, the by-word random intercepts for session were removed from the random-effects structure.

A statistically significant main effect of modality was only observed for occurrences 5 and 6 (β=1.691, t=-2.312, p=0.028), whereas no statistically significant difference between song and speech was observed for the other occurrence pairs (1vs2: β=-0.518, t=-0.637, p=.529; 3vs4: β=-0.502, t=-0.524, p=.604; 7vs8: β=-0.338, t=-0.435, p=.666).

D2. Occurrence-session interactions (supplement to Follow-up analysis #1 – development over eight familiarization occurrences)

The analyses of the development of the word recognition responses over the eight target occurrences within a familiarization passage revealed an interaction between the linear trend over occurrence and session for the model comprising both song and speech as well as the speech-only model.

To explore this interaction further, the development over occurrences was analyzed separately for sessions 1 and 2, with otherwise the same fixed effects as the main analysis, namely the first to third order polynomials for occurrence and the comparisons across the modalities (speech=-1; song=1) and the two time windows (250-500 ms=-1; 600-800 ms=1). We aimed for the same random-effects structure as in the first model addressing the development over familiarization occurrences (i.e., a by-subject random intercept and slope for modality as well as by-word random intercepts and slopes for modality and session). Due to convergence issues in both the session 1 and 2 models, only the by-subject and by-word random intercepts were included. Only the main effect of the linear trend and its significant interactions with other main effects will be reported.

The separate models for sessions 1 and 2 including both speech and the song data revealed a positive linear effect of occurrence that was statistically significant in session 2 (β=106.692, t=2.945, p=.003) and not in session 1 (β=-6.248, t=-0.151, p=.880). Note that slope in session 1 was even somewhat negative. The session 1 model also included a significant interaction between the linear trend and modality (β=82.825, t=1.998, p=.046), which will be further explored in the song-only and speech-only models.

The analysis was further broken down by separating between the song and speech data, with the random-effects structures necessarily including only by-subject and by-word random intercepts. The result patterns for sessions 1 and 2 as just reported were present in both the song and the speech data, with statistically significant positive linear effects of occurrence in session 2 (song: β=111.071, t=2.019, p=.044; speech: β=105.986, t=2.243, p=.025), and not in session 1 (song: β=77.346, t=1.266, p=.206; speech: β=-87.873, t=-1.567, p=.117). Not that the slope in session 1 was positive for song and negative for speech,

suggesting that the infants whose first session presented spoken materials might have displayed a somewhat negative-going word recognition response as opposed to infants in all other sessions.

D3. Formally establishing responder types (supplement to Additional follow-up analyses: Responder types in the test trials)

The key question in the second follow-up analysis is why no group-level effect was observed in the test phase. We specifically explored whether participants can be divided into Positive and Negative responders, based on the average amplitude difference between their responses to familiarized target words and novel control words in the test phase. Such an exploration of subgroups is common practice in the literature [69,70,71,93] - see also [67]- and if participants present a mix of Positive and Negative responders, a group-level absence of a clear effect might not reflect the absence of an effect for each individual infant.

In a first step towards making the division between Negative and Positive responders, we observed a strong correlation between participants' responses in the 250-500 ms and 600-800 ms time windows within each modality (song: r=.496, t(24)=2.797, p=.010; speech: r=.745, t(29)=6.014, p<.001; See Figure S1). This association motivated averaging over the time windows to obtain a more robust measure of each individual's response direction.

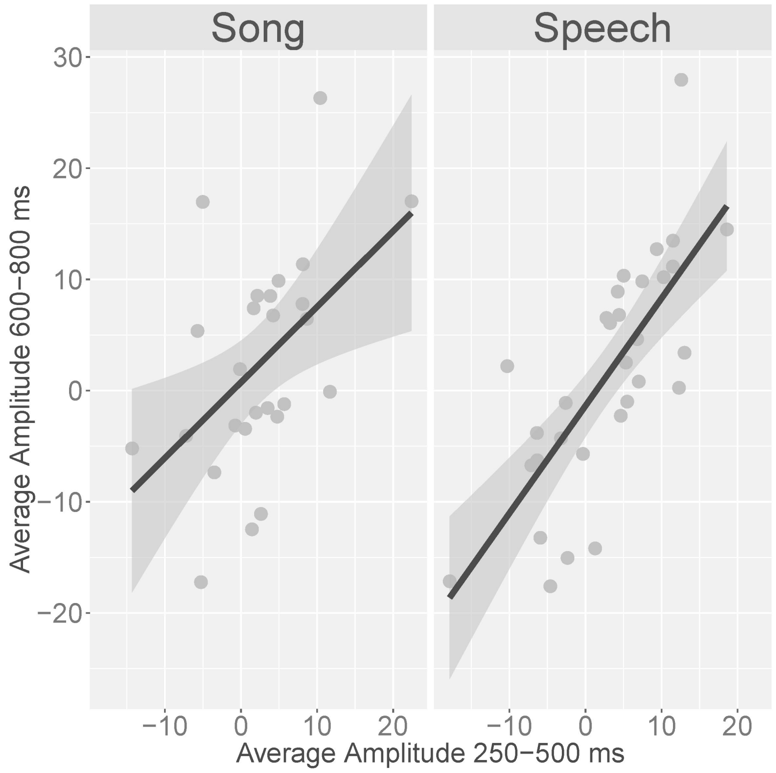

Figure S1. Association between the average EEG amplitudes (averaged over trials) in the 250-500 ms and 600-800 ms time windows in song (left panel) and speech (right panel). Grey points indicated participants' individual averages. The black lines with grey shadow provide the linear regression lines surrounded by 95% confidence intervals.

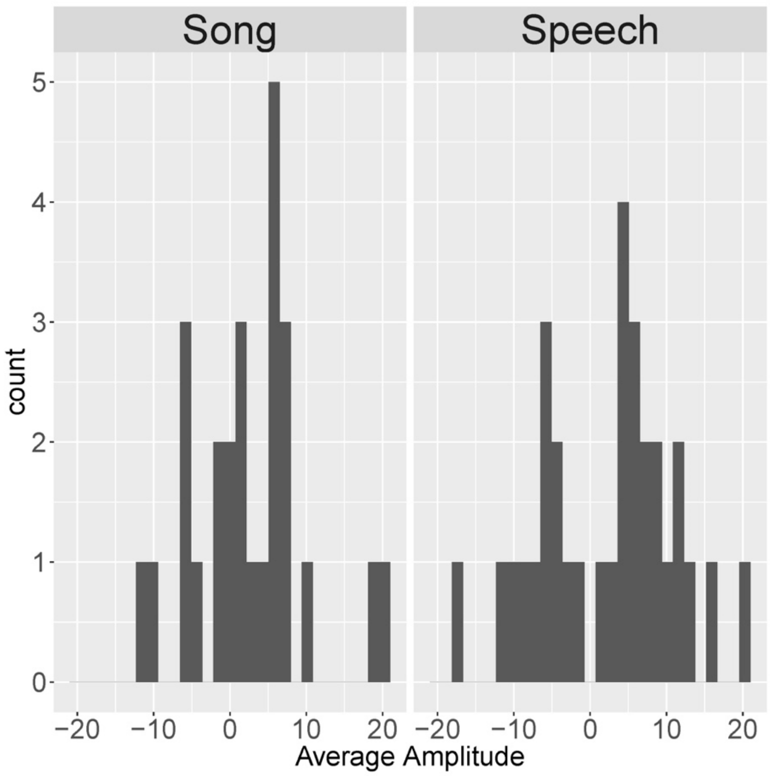

Figure S2 Histogram of participants' average amplitude in the test phase (averaged over trials and the 250-500 ms and 600-800 ms time windows) in Song (left panel) and Speech (right panel).

Visual inspection of the distribution of the children's responses (averaged over the 250-500 ms and 600-800 ms time windows) suggested the presence of two clusters in the speech data: one cluster with negative-leaning responses, the second with positive-leaning responses, and no infant having a difference score of exactly 0 (Figure 7, right). No such bimodality was apparent in the song data (Figure S2, left). These results present data-driven motivation for separating children in Positive and Negative responders based on a comparison between their familiarity response and zero, and we are looking forward to seeing this procedure replicated in future studies that divide their participants into such subgroups.

In a first attempt to formally assess the apparent bimodality in the data, separate Gaussian Mixture Models were fit to the word recognition effects in the song and the speech data (aggregated over the early and late windows), using *R* package *mclust*. Model comparisons using the Bayesian Information Criterion (BIC) favored a univariate normal mixture with one component for both the song and speech data (song: log-likelihood=-88.439; n=26; df=2; BIC=-183.394. speech: log-likelihood=-110.568; n=31; df=2; BIC=-228.004). In all cases, the next favored model was also a mixture with only one component. These results, therefore, do not provide strong support for the bimodality in the speech data.

As a second step to assess the bimodality in the data, we directly counted the number of local maxima in the kernel smoothed distributions of the one-dimensional song and speech data. Local maxima were detected using the *find_peak* function in the *R* package *ggpmisc*, which detects whether the value of a point along an axis is higher than both the preceding and the following value. The distributions were smoothed using 51 bandwidths ranging from 1 to 6 at 0.1-step intervals, in order to assess the stability of any pattern detected. Supplementary Figure S3 displays the number of peaks detected in both

datasets for each of the smooths, showing that the smooths of both the song and the speech data ultimately converge to a single peak and that two peaks are detected in each dataset for a total of 15 smooth kernel bandwidths. These results, again, do not provide strong support for stronger bimodality in the speech compared to the song data.

However, the bimodalities in the song and the speech data appear to be of a very different nature. This becomes apparent from Supplementary Figure S3, which displays the density distribution of the song and speech data at kernel bandwidths of 2, 3, and 5, in which three, two, and one peaks are detected, respectively. The two peaks detected in the speech data smoothed with a kernel bandwidth of 3 reflect the contrast between children with a word-recognition response below 0 (Negative responders, n=10) and those with a word-recognition response over 0 (Positive responders, n=15). In contrast, the detected bimodality in the song data separates children with a word recognition response below 10 versus above 18, with only two children in the latter cluster. These results are somewhat promising that a division into Negative versus Positive responders is warranted for the speech and not the song data.

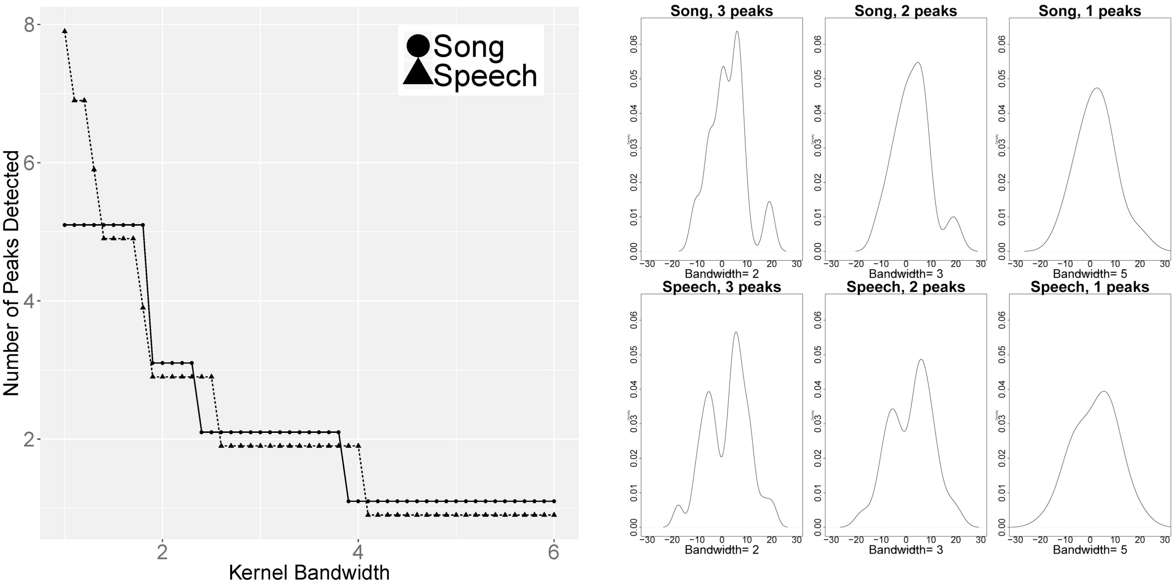

Figure S3. Left panel: Number of peaks detected (y-axis) in kernel-smoothed data with a range of bandwidths (y-axis) in the Song data (circles, dotted line) and the Speech data (triangles, solid line). Right panel: Kernel-smoothed data in Song (top) and Speech (bottom) with hand-selected kernel bandwidths of 2, 3, and 5.

These analyses highlight the difficulty in establishing bimodality in small data sets and should be seen as caution against strongly interpreting the difference between Negative and Positive responders. Yet, the widespread in responses suggests that the group-level null effect in the test phase may mask a split between Negative and Positive responders and thus a robust segmentation effect.

D4. Experimental control and responder types (supplement to Additional follow-up analyses: Responder types in the test trials)

After having classified participants as Negative or Positive responder in song and speech separately, we assessed to what extent response types were associated across the modalities for the 25 participants who contributed test data to both sessions. Although inspection of the raw numbers suggested a

tendency for the positive response type to be stable across the two conditions (Supplementary Table S6), this was not supported by a McNemar's test for independence with a continuity correction ($\chi(1)=0$, $p=1$). Moreover, there was no evidence that the word recognition responses were associated between the modalities for the 25 participants who contributed data for both sessions ($r=-0.005$, $t(23)=-0.026$, $p=.979$). It was therefore decided to analyze the responder types separately between the conditions.

Table S6: Association table between participants' classified Responder Type in the Speech and Song modality, for the 25 infants contributing data to the experimental sessions in both modalities.

			Song Responder Type	
			Positive	Negative
Speech	Responder Type	Positive	10	5
		Negative	6	4

Before proceeding to use Responder Type as an independent variable is subsequent analyses, it was critical to establish whether they might be a side effect of experiment variables or the infant's engagement in the familiarization phase.

To assess whether the Negative and Positive responders were skewed across the modalities, sessions, or versions, we conducted a mixed-effects logistic regression model with responder type as the dependent variable (Positive = 1, Negative =0), and fixed independent variables modality (contrast coded, Speech=-1; Song=1), session (contrast coded, Session 1 = -1, Session 2 = 1), and version (deviation coded). Modality and Session interacted, Version was only entered as a main effect due to convergence problems. The random effects component of the model only included a random intercept by subject. The only (marginally) significant effect was a Modality by Session interaction (β =0.594, $z=-1.893$, $p=.0583$), reflecting a strong skew to positive responders in Session 2 of the Speech condition only. Importantly, there was no strong indication that the positive and negative responders were unequally distributed across the versions.

D5. Comparing development in the familiarization phase across responder types in the test trials (supplement to Additional follow-up analysis: Responder types in the test trials)

The final follow-up analysis assessed whether the development across target word occurrences in the speech familiarization passages differed between Negative and Positive responders. Visual inspection of the data suggested that the sinusoid-shaped development was present in both subgroups, yet more pronounced for Negative compared to Positive responders (see Supplementary Figure S4). This was supported by the statistical analyses, only finding evidence for the sinusoid-shape in the Negative responders.

The main model to assess this was a linear mixed-effects model with the independent variable response type (contrast coded, Negative = -1; Positive=1) in addition to the three occurrence polynomials and time window contrasts discussed earlier. For consistency with the first follow-up analysis, the random-effect structure included by-subject and by-word random intercepts as well as a by-word random slope for Session. From the three polynomial trends included, only the cubic trend was

significant (β=141.891, t=3.812, p<0.001) and marginally significantly modulated by the Responder Type (β=-67.033, t=-1.801, p=0.072). No other effects were statistically significant or marginally significant. Because of the interaction between the cubic trend and Responder type, interpretation of these results requires subset analyses of the Negative and Positive responders.

These subset analyses had the same random-effects structure as described above. The cubic trend was significant for the Negative responders (β=211.703, t=3.565, p<0.001) and not for the Positive responders (β=71.894, t=1.583, p=.114). Note that the estimate for the Positive responders was positive and about half the size of the estimate for the Negative responders. This result confirms the visual inspection of Supplementary Figure S4, namely that the sinusoid-shaped development across speech blocks is more pronounced for Negative than Positive responders.

The outcomes from this final follow-up analysis strengthen the conclusions from both the first and the second follow-up analyses. First, these outcomes show that the sinusoid-shaped development over occurrences of target words in the familiarization phase is modulated by the participants' Responder type, a feature that has been established outside of the familiarization phase. Second, the difference between Negative and Positive responders during test is shown to have implications for their behavior in the preceding familiarization phase as well. Combined, these three follow-up results suggest that infants with a more mature negative response in the test phase showed a stronger modulation over the familiarization phase than the infants with a less mature positive response.

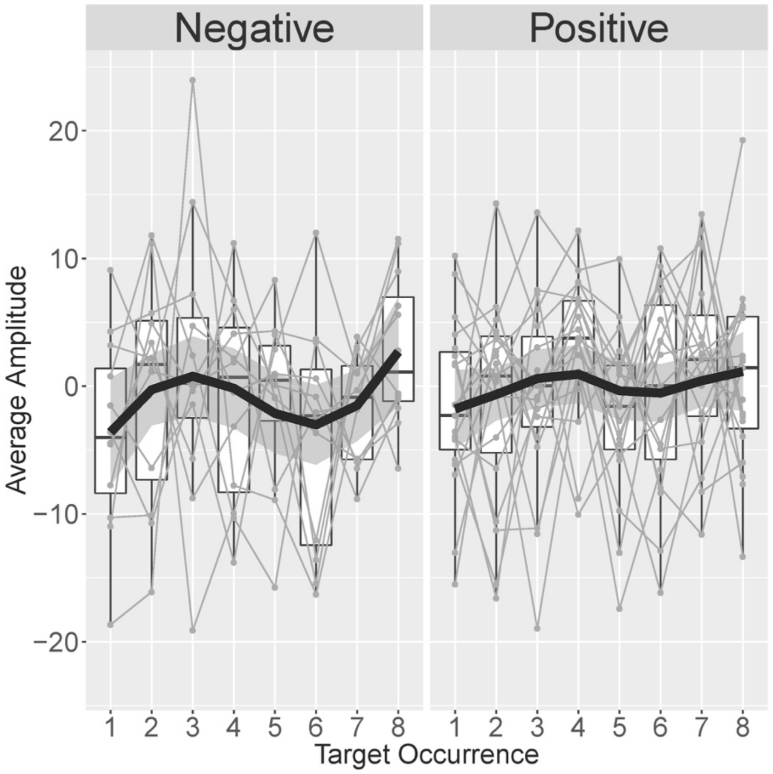

Figure S4. Average EEG amplitude in μV (averaged over blocks) in the 250-500 ms time window across the eight target occurrences in the familiarization phase in speech in Negative Responders (left panel) and Positive Responders (right panel). Grey lines connect individual participants' averages, indicated by grey points. The black lines provide a LOESS-smoothed development averaged over participants.
